# Modulation of stress and immune response by Amblyomin-X results in tumor cell death in a horse melanoma model

**DOI:** 10.1038/s41598-020-63275-2

**Published:** 2020-04-14

**Authors:** Flavio Lichtenstein, Asif Iqbal, Sonia Elisabete Alves de Lima Will, Rosemary Viola Bosch, Carlos DeOcesano-Pereira, Mauricio Barbugiani Goldfeder, Roger Chammas, Carlos Eduardo Madureira Trufen, Katia Luciano Pereira Morais, Jean Gabriel de Souza, Renato Jose Mendonça Natalino, Inacio Junqueira de Azevedo, Milton Yutaka Nishiyama Junior, Ursula Oliveira, Francisco Ivanio Arruda Alves, Jaqueline Mayara Araujo, Aline Ramos Maia Lobba, Ana Marisa Chudzinski-Tavassi

**Affiliations:** 1 Laboratory of Molecular Biology, Butantan Institute, São Paulo, SP Brazil; 2 CENTD, Centre of Excellence in New Target Discovery, Butantan Institute, São Paulo, Brazil; 3 ICESP, Center for Translational Research in Oncology, Instituto do Câncer do Estado de São Paulo, Faculdade de Medicina da Universidade de São Paulo, São Paulo, Brazil; 4 Laboratório Especial de Toxinologia Aplicada - CeTICS, Butantan Institute, São Paulo, Brazil

**Keywords:** Bioinformatics, Melanoma, Cellular signalling networks, High-throughput screening, Translational research

## Abstract

We have investigated Amblyomin-X-treated horse melanomas to better understand its mode of action through transcriptome analysis and the *in vivo* model. Amblyomin-X is a Kunitz-type homologous protein that selectively leads to the death of tumor cells via ER stress and apoptosis, currently under investigation as a new drug candidate for cancer treatment. Melanomas are immunogenic tumors, and a better understanding of the immune responses is warranted. Equine melanomas are spontaneous and not so aggressive as human melanomas are, as this study shows that the *in vivo* treatment of encapsulated horse melanoma tumors led to a significant reduction in the tumor size or even the complete disappearance of the tumor mass through intratumoral injections of Amblyomin-X. Transcriptome analysis identified ER- and mitochondria-stress, modulation of the innate immune system, apoptosis, and possibly immunogenic cell death activation. Interactome analysis showed that Amblyomin-X potentially interacts with key elements found in transcriptomics. Taken together, Amblyomin-X modulated the tumor immune microenvironment in different ways, at least contributing to induce tumor cell death.

## Introduction

Melanoma is a type of cancer arising from the malignant transformation of melanocytes, pigment producing-cells found predominantly in the basal layer of the epidermis and eyes. Cutaneous melanoma is the most aggressive and treatment-resistant form of skin cancer responsible for the vast majority of skin cancer-related deaths in the Caucasian population^1^. The global incidence of melanoma continues to increase at an alarming rate, despite decades of public prevention programs in many countries. Around 232,000 new cases of skin cancer were recorded worldwide in 2012, accounting for 1.6% of all new cases of cancer back then, while over 300,000 new cases of melanoma were diagnosed worldwide in 2018, according to the World Cancer Research Foundation^2,3^. In Brazil, 1,547 deaths were recorded in 2013 due to melanoma, with around 5,690 new cases reported back then, while around 6,260 new cases were expected due in 2018, according to the National Cancer Institute (INCA)^4^.

Cutaneous melanoma usually affects a higher proportion of patients, in the age range 40–60 years. They can be treated by surgical excision when detected in the early stage (0, I, II and resectable III), however, in the later stages (unresectable III, IV and recurrent melanoma) the treatment options are chemotherapy, target therapy (BRAF/MEK pathway inhibitors), immunotherapy (checkpoint blockade CTLA-4 receptor inhibition, PD-1 ↔ PD-L1 axis inhibition, and interferon-gamma immunotherapy), or a combination of them. Death in most patients is caused by metastatic disease which may have evolved from the primary tumor. Therefore, there is a need for new strategies in melanoma therapy because many patients do not respond to these treatments due to mutations in their genome, microbiota profile, response to neo-antigens, and resistance to drugs^5^.

In horses, skin tumors are the most common among neoplasms. Horse melanomas are encapsulated complex systems surrounded by a hard peel and represent 5 to 14% of equine skin neoplasms^6,7^. These melanomas are usually benign tumors; however, they may have an unpredictable malignancy, progressing to malignant forms, and metastasize^6^. Dark-haired horses feature higher melanoma malignancy as they get older compared to white-haired horses^7^.

Malignant transformations are caused by driver mutations. The acknowledged mutated genes in human melanoma are *BRAF* and *NRAS*, as well as the newly discovered *PPP6C*, *RAC1, SNX31, TACC1, STK19*, and *ARID2*^8^. In horses, the only recognized driver mutation hitherto is caused by a 4.6 Kb duplication in intron 6 of Syntaxin 17 (*STX17*)^9,10^^.^ As is known, spontaneous horse melanoma tumors have a different natural history and many anatomical differences, compared to human melanoma tumors, but for some^11,12^ it is considered a good translational model.

In the present study, we treated horse melanoma tumors with Amblyomin-X, a Kunitz-type recombinant protein identified in a cDNA library from the tick *Amblyomma sculptum* salivary glands^13^. This molecule has the ability to inhibit Factor Xa in the blood coagulation cascade and triggers apoptosis by activating its intrinsic pathway in tumor cells^14–16^. In previous studies, Amblyomin-X showed more avidity in the recognition of tumor cells, and rapid excretion in healthy murines^17^. We have already shown that Amblyomin-X causes cell death via induction of both endoplasmic reticulum (ER) stress and proteasome inhibition (PI) in renal adenocarcinoma (murine RENCA), in melanoma (murine B16F10 and human SK-MEL-28)^18^, as well as in pancreatic (human Mia-Paca-2) tumor cell lines^17,19,20^.

Over the past ten years, preclinical studies have shown the potential of proteasome inhibition as an effective therapeutic strategy for treating different types of cancers^21–24^ including melanoma^25–29^. Bortezomib and Amblyomin-X are proteasome inhibitors (PIs) activating different innate immune pathways, the former via MAVS-NOXA^25^, and the latter via RIG-I-MAVS, as suggested by our transcriptome analysis. Trials with Bortezomib^30–35^ alone have shown insufficient clinical efficacy, and in combination with paclitaxel and carboplatin, limited clinical benefit and significant toxicity^30^. Therefore, the effort to study proteasome inhibitors in different cancer therapies progresses^31,36^.

Herein, we investigated the effect of Amblyomin-X intratumoral injection in horse melanomas, followed by interactome and transcriptome analyses conducted to understand genes modulation and respective enriched pathways. The data suggest that Amblyomin-X modulates several pathways responsible for tumor cell death and its immune microenvironment. Moreover, transcriptome analysis suggests potentially yet unknown DEGs and novel target pathways, mainly in relation to the responses of the immune system.

## Results

### Equine melanoma regression upon treatment with Amblyomin-X

**Figure 1:**
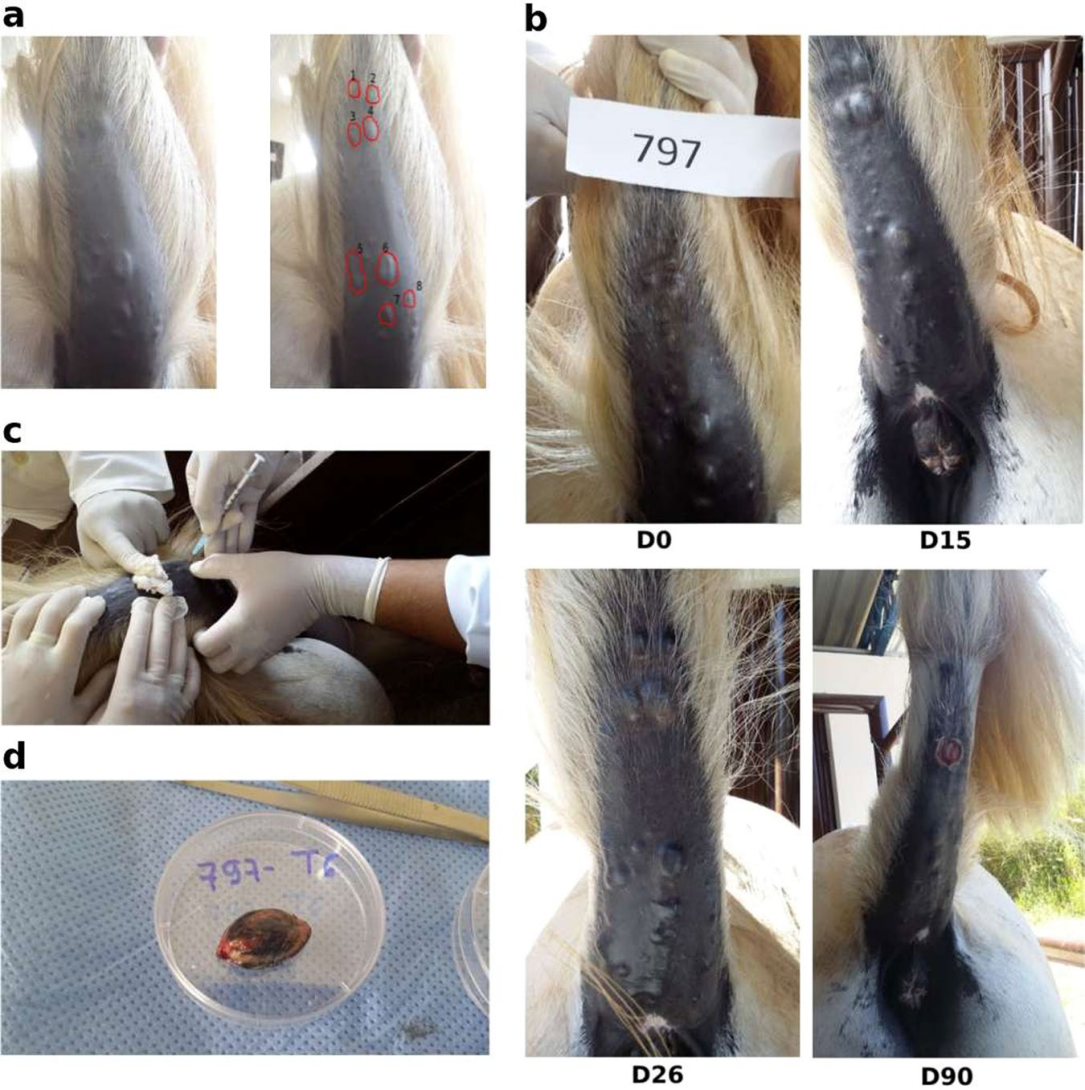
*In vivo* animal 797 tumor treatment and excision. We can see on the left (**a**) animal 795 tail and chosen tumors: 1 and 8 as control, 2, 3 and 5 as vehicle, 4, 6, and 7 as treatment; (**b**) horse tail and tumor evolution on day 0, 15, 26 and 90 (excision); (**c**) veterinarian injecting Amblyomin-X solution or PBS in a tumor; and (**d**) T6, one of the excised tumors. Tumors were chosen related to distance and size. Three tumors, with a diameter of around 1.5 cm, were chosen for treatment. About three farthest tumors were chosen as vehicle and other three tumors as control, whenever possible.

**Figure 2:**
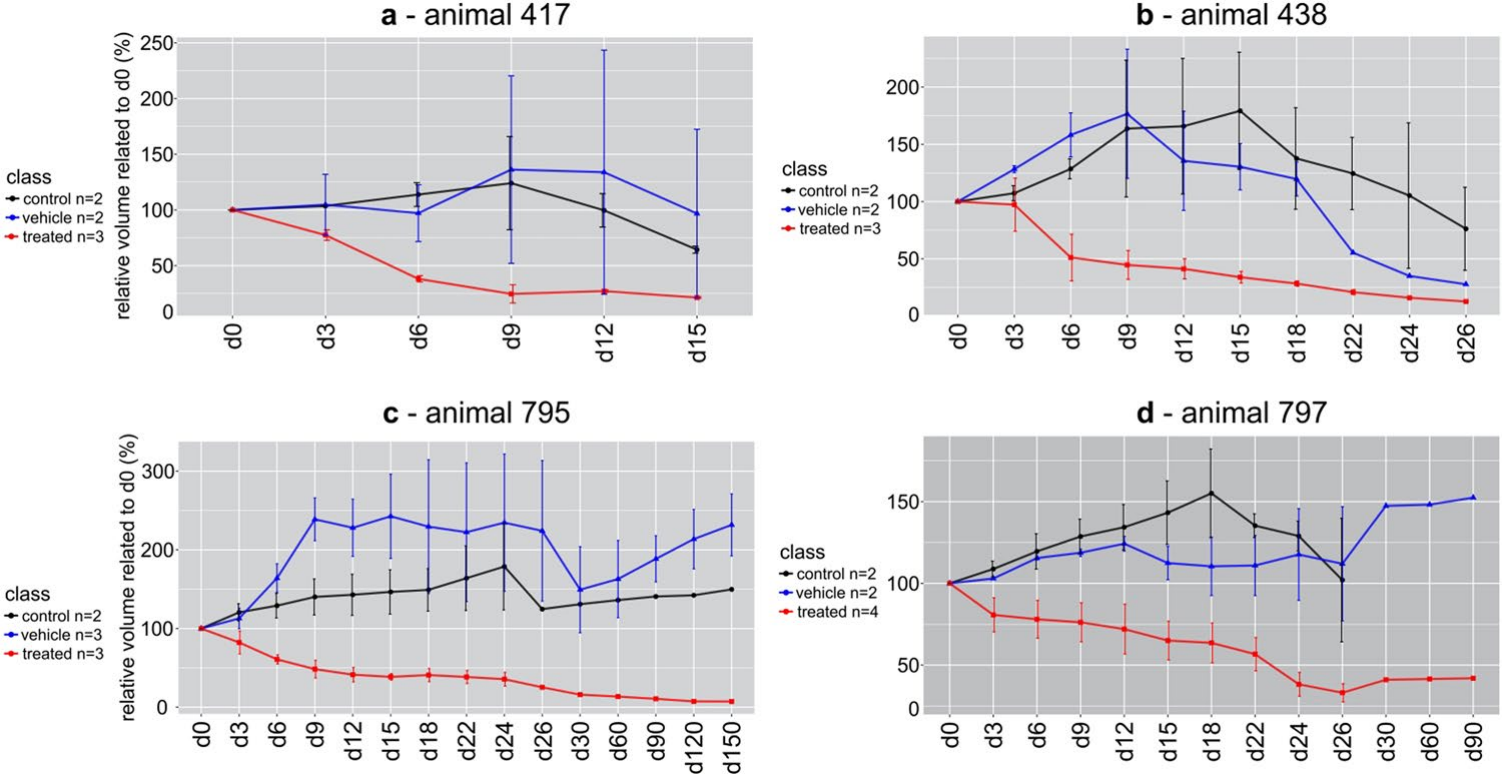
This plot shows the percentage volume curves related to the initial volume (0 h). Every three days, Amblyomin-X was injected for about one month’s time. Animals 795 and 797 and their tumors were followed for at most 5 months. Treated tumor volume curves were colored red, control tumor volumes green, and vehicle tumor volumes blue, for animals 417 in (**a**), 438 in (**b**), 795 in (**c**) and 797 in (**d**). Each treated curve shows a decrease in tumor volumes. Vehicle and control animals also showed a decrease in tumor volumes for animals 417 and 438. Animals 795 and 797 showed either a constant or increased volume related to the initial volume. Since each tumor has its own history and fate, the rate of decrease is unpredictable.

We injected Amblyomin-X intratumorally, at 1 mg/kg of the tumor mass, on each third day, during 28 days, and followed the tumor volume evolution and clinical animal conditions by over a period of 5 months. We treated some selected tumors in the ventral tail of four animals by following three groups: treated (Amblyomin-X), and two control groups, vehicle (PBS) and untreated (see Materials and Methods). Tumor dimensions were recorded before each injection and calculated the relative volume means related to the initial volume (Fig. 1). In all cases Amblyomin-X treatment promoted a reduction of at least 75% in tumor volume at the end of the treatment period (one month). Tumor #5 (from animal number 417) completely disappeared after 12 days under treatment, while tumors from another animal (number 795) continued to show volume reduction, even one month after the end of treatment. Interestingly some animal control tumors (untreated and PBS-injected vehicle), that were in proximity to tumors treated with Amblyomin-X, also showed regressions in tumor size, which might suggest an indirect effect that could be related to the lymphatic system or recruitment of the immune system cells. In two other animals, control tumors maintained or increased their initial volumes. The evolution curves of each tumor per animal are shown in Fig. 2 (see also, Table S1). It is worth to mention, that each horse tumor is presented as an independent capsule. We believe that long-term development and shrinkage are similar to all tail horse melanoma tumors, but experiments showed that each tumor responds differently, and we don’t know the reason.

### Clinical evaluation and biochemical parameters

Global biochemical parameters *i.e*. urea, creatinine, TGO (AST, aspartate aminotransferase), GGT (gamma-glutamyl transferase), BT (total bilirubin), BD (direct bilirubin), BI (indirect bilirubin), FAL (alkaline phosphatase) and ALB (Albumin) were all within normal limits during and after treatment termination. We have observed no adverse effects in the animals used in our study (Table S2).

### Histological analysis

**Figure 3:**
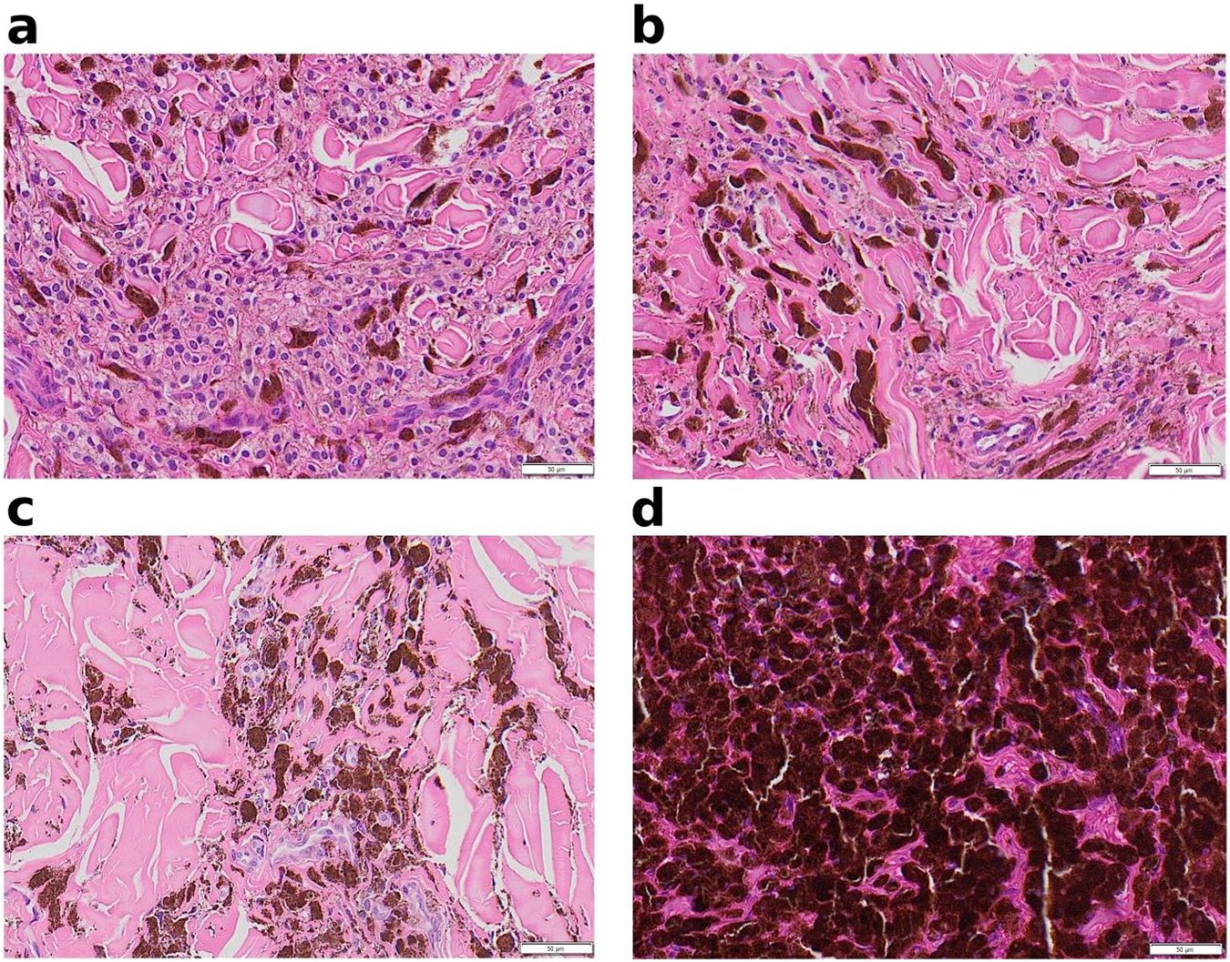
*In vivo* histology. Distinct histopathological slides are presented. (**a**) animal 438, tumor 2, vehicle excised on day 28 – an untreated tumor showing melanophages and many atypical melanocytes. (**b**) and (**c**) animal 797, tumor 3, vehicle excised at day 28 – an untreated tumor showing less atypical melanocytic cells. (**d**) animal 797, tumor 6, treated with Amblyomin-X and excised at day 28 – regression areas showing only melanophages can be seen.

Histological analysis of untreated and treated tumors showed proliferation of atypical and hyperpigmented melanocytes, presence of numerous macrophages phagocytizing melanin, but only a few perivascular lymphocytes (Fig. 3). Tumor treated for 28 days (animal 797, tumor 6), showed regression areas represented by the absence of atypical melanocytes, only remaining melanophages (Fig. 3d).

### Bioinformatics

#### Mapping and differential expression

Alignment and read count resulted in 269,991 transcripts, displayed in a matrix with horse Ensembl IDs as rows and 18 samples as columns, of which we removed two with low library size, both related to 12 h samples. We also excluded lowly expressed genes. We used edgeR to calculate the normalized expression table in CPM and BioMaRt to find horse-to-human ortholog coding genes, which yielded 14.414 transcripts, of which 13,138 transcripts had valid gene symbols for horse and 13,943 for human. There were more symbols for human genes due to paralog-ortholog conversion. For horse transcripts, there were 546 DEGs for 6hx0h and 259 DEGs for 12hx0h (Table S3). Those DEGs having human orthologs were 546 for 6hx0h and 259 for 12hx0h. We could not find DEGs for the 12hx6h comparison, probably because gene expressions between these time points are somehow very close.

#### Pathway analyses

Metacore pathway enrichment analysis found 196 pathways for 6hx0h and 67 pathways 12hx0h. The same analysis can be seen using Reactome, String-db with KEGG, and String-db with GO Biological Processes (Table S4).

We classified the enriched pathways according to the words in each pathway description name that may represent a function or have a gene symbol (Fig. S1a). We also ranked these pathways regarding how strongly modulated they were (delta of the sum of LFC = sum of LFC for 6hx0h minus sum of LFC for 12hx0h, for all genes in the pathway), resulting in positive values for pathways more strongly modulated for 6hx0h compared to 12hx0h, negative if more strongly modulated for 12hx0h compared to 6hx0h, and similar if the modulations were close to each other (Table S5). For instance, the Innate Immune System (IIS) class comprised 26 pathways, of which 19 had genes more strongly modulated for 6hx0h, 3 for 12hx0h, and 4 were equally modulated (Fig. S1b–d). According to the pathway classification, we have found that “immune system” was the class with more enriched pathways (80 pathways), according to Metacore, followed by “signaling” (55 pathways), “lung” (37 pathways), “canonical pathway” (31 pathways), “innate immune system” (26 pathways), “inflammation” (23 pathways), “cancer” (22 pathways), “muscle” (18 pathways), and “adhesion/ECM/cytoskeleton” (15 pathways).

Figure S1b shows 6hx0h higher modulated pathway classes, where immune system, pathway signaling, (lung) inflammation, innate immune system, canonical pathway, cancer, disease, migration/invasion/motility, adhesion/ECM/cytoskeleton, apoptosis, muscle, immunogenic cell death, virus responses, and stress, stand out as the most important pathway classes for this condition.

Figure S1c shows 12hx0h higher modulated pathway classes, however only a few pathways can be seen such as adhesion/ECM/cytoskeleton, lung, muscle, immune system, receptor pathways, signaling pathways, innate immune system, migration/invasion/motility, and cancer, resulting in a weaker response for 12hx0h when compared to 6hx0h.

Finally, fig. S1d shows classes with similar responses for 6hx0h and 12×0h, including immune system, pathway signaling, canonical pathways, development, complement, muscle, cancer, dermis, and innate immune system.

In summary, most of the pathways were related to cell stress, innate immune response/inflammation, cytokines, cell damage/endothelial, neutrophil, apoptosis, ECM, cell adhesion, muscle, T-help, complement, and coagulation. Therefore, pathway classes were divided into three groups: a) first responses (ER-stress and IIS); b) confounding factors; and c) secondary responses (Table 1). Herein, confounding factors are denoted as enriched pathways, and part of the expression of their modulated genes, observed not probably due to the drug action, but mainly due to the stress and wounds caused by the injections. Next, we summarize the results highlighting pathways. In the text below, all pathway classes are written in bold.

**Table 1:** Selected enriched pathways that represent genes and important functions related to the *ex vivo* experiment.

a – first responses			
**Functional**	**Pathway**	**DEGs(6hx0h)**	**DEGs(12hx0h)**
ER-stress	HCV-mediated liver damage and predisposition to HCC via cell stress	CALR, CASP3, CYCS, HSP90B1, HSPA5, HSPD1, IL1R1, IL6, IL6ST, ITPR1, SOD2, STAT3, XBP1	
	Endoplasmic reticulum stress response pathway	CYCS, HSP90B1, HSPA5, ITPR1, SOD2, XBP1	
	Role of PKR in stress-induced antiviral cell response	CASP3, EIF2AK2, IL1B, IL1R1, IL6, IL8, STAT1, TLR2	EIF2AK2, IL1B, IRF7, STAT1, TLR2
TLR (IIS)	HSP60 and HSP70 - TLR signaling pathway	HSPA5, HSPA6, HSPA8, HSPD1, IL1B, IL6, IL8, TLR2	
RLR (IIS)	Innate immune response to RNA viral infection		DDX58, DHX58, IFIH1, IRF7
OSM (IIS)	Oncostatin M signaling via JAK-Stat	IL6ST, OSMR, SERPINA3, SOCS3, STAT1, STAT3, TIMP1	SOCS3, STAT1, TIMP1
	Oncostatin M signaling via MAPK	IL6ST, LDLR, MMP3, OSMR, STAT1, TIMP1	
OAS/RNase L (IIS)	Antiviral actions of Interferons	EIF2AK2, IRF9, OAS2, STAT1, WARS	EIF2AK2, IRF9, MX1, OAS1, OAS2, STAT1
**b – confounding factors**			
**Functional**	**Pathway**	**DEGs(6hx0h)**	**DEGs(12hx0h)**
Neutrophil	Inhibition of neutrophil migration by proresolving lipid mediators in COPD	ACTN2, C5AR1, CD34, CXCR2, FPR2, IL1B, IL1R1, IL8, ITPR1, PRKCQ, PTAFR, TLR2	ACTN2, CXCR2, IL1B, PRKCQ, TLR2
Eosinophil	Eosinophil chemotaxis in asthma		C3, CCL7, CXCL10, CXCR2, NGF
Cell adhesion	ECM remodeling	IGFBP4, IL8, MMP3, PLAUR, SERPINE1, TIMP1	
	Integrin inside-out signaling in neutrophils	CXCL1, CXCR2, IL8, ITPR1, LYN, PTAFR, RASGRP2, SELE, SELP	
Muscle	Muscle contraction: Delta-type opioid receptor in smooth muscle contraction	ITPR1, MYH8, PENK	MYH8, PENK
**c – secondary responses**			
**Functional**	**Pathway**	**DEGs(6hx0h)**	**DEGs(12hx0h)**
Inflammation	Release of pro-inflammatory factors and proteases by alveolar macrophages in asthma	CXCL1, IL1B, IL6, IL8, MMP3, STAT1, TIMP1, TLR2	CXCL10, IL1B, STAT1, TIMP1, TLR2
	Release of pro-inflammatory mediators and elastolytic enzymes by alveolar macrophages in COPD	CTSL, IL1B, IL6, IL8, STAT1, TLR2	CTSL, CXCL10, IL1B, STAT1, TLR2
	Inflammatory mechanisms of pancreatic cancerogenesis	CD46, CXCL1, CXCR2, IL1B, IL1R1, IL6, IL8, STAT1, STAT3	
IL-1	IL-1 signaling pathway	BIRC3, CCL7, CXCL1, IL1B, IL1R1, IL6, IL8, PTGES, STAT1, ZC3H12A	
	The innate immune response to contact allergens	HSPA5, HSPA6, HSPA8, IL1B, IL1R1, IL6, IL8, TLR2	
Cell migration	Chemotaxis: CCL16-, CCL20-, CXCL16- and CCL25-mediated cell migration	ITPR1, MMP11, MMP3, MMP8	
Cytoskeleton remodeling	Cytoskeleton remodeling: Regulation of actin cytoskeleton organization by the kinase effectors of Rho GTPases	ACTN2, MYH8	ACTN2, MYH8
Apoptosis & Survival	Endoplasmic reticulum stress response pathway	CYCS, HSP90B1, HSPA5, ITPR1, SOD2, XBP1	
	Apoptosis and survival: CXCR3-B signaling	CASP3, CYCS, HMOX1, ITPR1, RYR1, STAT3	
	Apo-2L(TNFSF10)-induced apoptosis in melanoma	CASP3, CASP4, CYCS, IL8, XBP1	
Interferon-alpha/beta	IFN-alpha/beta signaling via JAK/STAT	EIF2AK2, IFIT3, IL1RN, IRF9, ISG15, SOCS3, STAT1, STAT3	CXCL10, EIF2AK2, IFIT3, IFITM1, IL1RN, IRF7, IRF9, ISG15, ISG20, OAS1, SOCS3, STAT1, XAF1
	IFN-alpha/beta signaling via MAPKs	EIF2AK2, IFIT3, IRF9, ISG15, PRKCQ, STAT1, ZBTB16	CXCL10, EIF2AK2, IFIT3, IRF7, IRF9, ISG15, PRKCQ, STAT1

The four columns are the functions, pathways, enriched DEGs for 6hx0h comparison and for 12hx0h comparison, respectively. The table was divided into A, B, and C, for pathways classified as “ER-Stress and IIS”, “confounding factors”, and “secondary responses”, respectively.

**ER-stress** was inferred due to the enriched pathways such as “HCV-mediated liver damage and predisposition to HCC via cell stress”, “Endoplasmic reticulum stress response pathway”, and “Role of PKR in stress-induced antiviral cell response”. For **Immune System**, six innate immune system responses were found: TLR pathway, RIG-I-like Receptors (RLR), Oncostatin-M pathway, OAS/RNase L pathway, Neutrophil, Eosinophil pathways.

**TLR** was inferred due to the enriched “HSP60 and HSP70 - TLR signaling pathway”; **RLR** due to the enriched “Innate immune response to RNA viral infection” pathway; and **Oncostatin-M** due to the enriched “Antiviral actions of Interferons” pathway.

For the present study, **Neutrophil, Eosinophil** and **Complement** are termed as confounding factors, since these biological functions are related to the damage caused by the intratumoral injections, activating “endothelial damage” and “wound healing” pathways. Another confounding factor is **Muscle contraction**, to be discussed later. All data related to these pathways can be seen in Table 1B.

In addition, **ER-stress** and **Innate Immune Responses** should activate many secondary responses like Inflammation, IL-1, Cell migration, Cytoskeleton remodeling, Apoptosis & Survival, and Interferon-alpha/beta. **Inflammation** was inferred due to the enriched pathways related to macrophages: “Release of pro-inflammatory factors and proteases by alveolar macrophages in asthma”, “Release of pro-inflammatory mediators and elastolytic enzymes by alveolar macrophages in COPD”, “Inflammatory mechanisms of pancreatic cancerogenesis”, “Immune response_IL-6-induced acute-phase response in hepatocytes”, and “Immune response_TREM1 signaling pathway”; and **IL-1** was inferred due to enriched pathways: “IL-1 signaling pathway” and “The innate immune response to contact allergens”.

Moreover, **Cell Migration** was inferred due to the enriched “Chemotaxis: *CCL16*-, *CCL20*-, *CXCL16*- and *CCL25*-mediated cell migration” pathway; and **Cytoskeleton Remodeling** was inferred due to the enriched “Cytoskeleton remodeling: Regulation of actin cytoskeleton organization by the kinase effectors of Rho GTPases” pathway. Finally, **Apoptosis & Survival** was inferred due to enriched “Endoplasmic reticulum stress response pathway”, “Apoptosis and survival: CXCR3-B signaling”, and “Apo-2L(TNFSF10)-induced apoptosis in melanoma” pathways; and **Interferon-alpha/beta** was inferred due to the enriched “Immune response: IFN-alpha/beta signaling via JAK/STAT” and “Immune response: IFN-alpha/beta signaling via MAPKs” pathways.

### Network analyses

**Figure 4:**
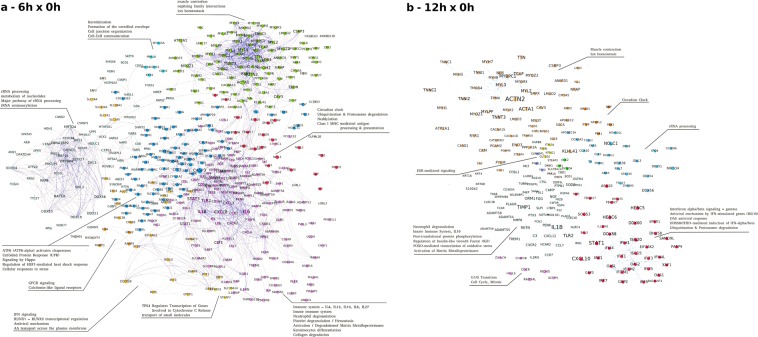
Network analysis. Gephi clusterized DEGs according to the modularity index with resolution of 1.2 and using weights, and respective enrichment analyses were done using Reactome. On the left (**a**) are all DEGs related to 6hx0h comparison, and 9 clusters could be defined related to Immune System, Transport & Cytochrome C, Antiviral pathways (RLR), GPCR pathway, UPR, rRNA processing & metabolism of nucleotides, Cell junction & cell-cell communication, muscle & ion homeostasis, and Circadian pathway & Ubiquitination. On the right (**b**) are DEGs related to 12hx0h comparison, resulting in a smaller network, with seven clusters: Innate Immune System responses, Cell cycle pathways, Neutrophil Degranulation, ESR-mediated signaling, Muscle, Circadian Clock, and rRNA processing.

With the application of String-db, 93 enriched pathways for 6hx0h and 59 for 12hx0h were found. String-db allows for the calculation of protein-protein interaction (PPI) networks, and with igraph^37^ it was possible to figure out the connectivity index (k) and betweenness centrality (g) (Table S6). Next, Gephi was used to cluster genes according to the modularity index (community detection algorithm) with a resolution of 1.2 and the use of weights. The result can be seen in Table S7 and respective networks can be seen in Fig. 4, where the main enriched pathways could be achieved using all clustered DEGs and Reactome to find the enriched pathways. Node colors were obtained using modularity classes for nodes clusterization, their sizes are proportional to the degree of connectivity and the label size is proportional to the betweenness centrality.

### *In-silico* crosstalk simulation

#### Crosstalk between pathways

Five different possible crosstalks were simulated: a) adhesion-ECM-cytoskeleton versus remodeling versus stress, b) ICD versus apoptosis versus ER-chaperone-Golgi, c) cancer versus complement versus inflammation, d) IIS versus apoptosis” versus autophagy, and e) angiogenesis-vascular versus hypoxia versus stress. For this purpose we used Circlize R package.

**Figure 5:**
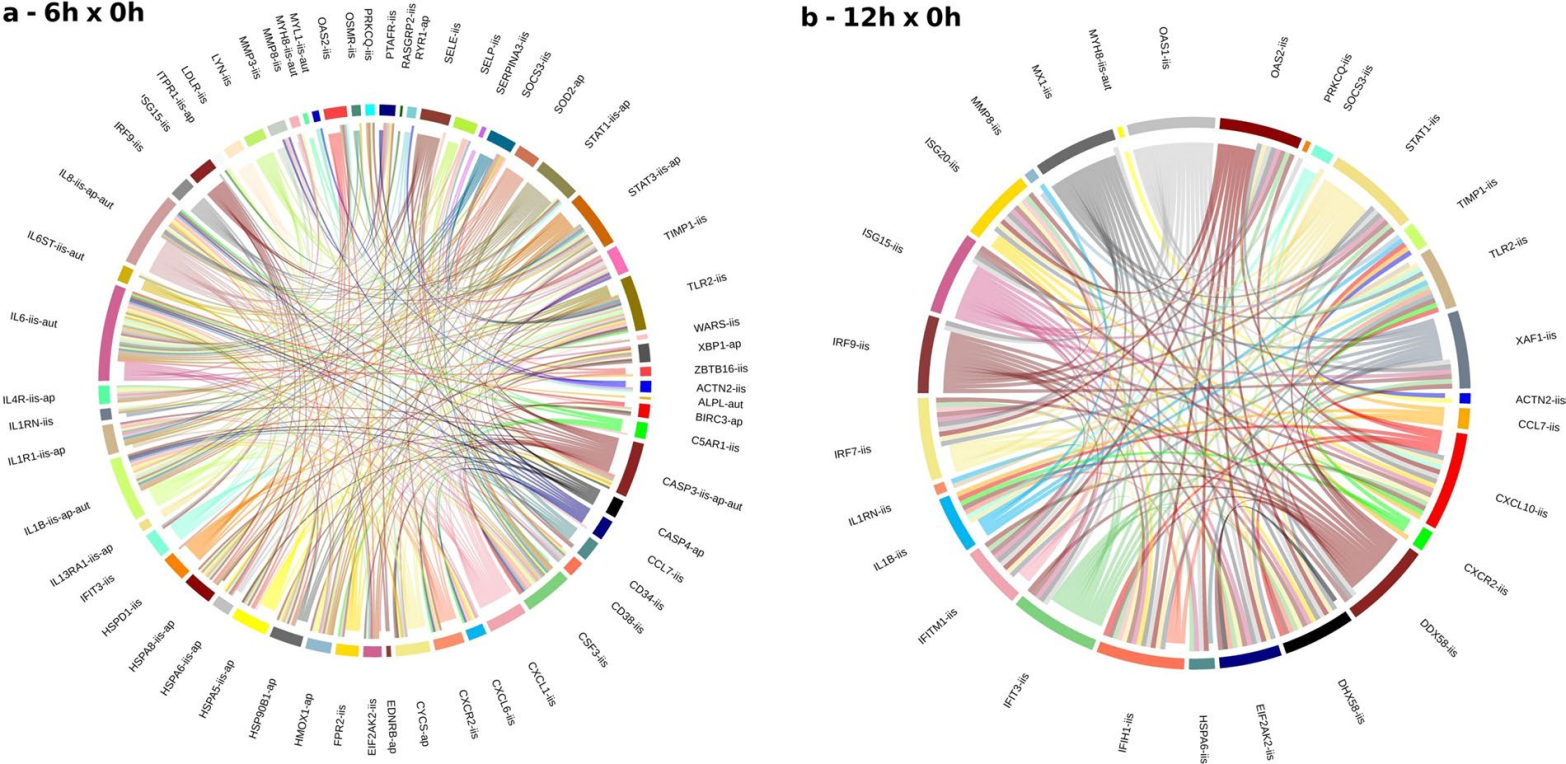
Crosstalk between three pathway classes. To better visualize the crosstalk between DEGs, we downloaded the String-db PPI table and filtered only interactions with scores greater than or equal to 0.4. On the left (**a**) is the crosstalk between DEGs related to innate Immune system, apoptosis and autophagy, with all 59 DEGs well connected, which leads to the inference that IIS and Apoptosis have common genes to work properly, or to be activated. Only autophagy was a less significant pathway, not observed until 12 h by transcriptome analysis. We can see on the right (**b**) all 28 DEGs for 12hx0h, with fewer genes and interactions comparing to 6hx0h. Near each gene are its possible pathways like ‘iis’ (innate immune system), ‘ap’ (apoptosis), and ‘aut’ (autophagy). In supplementary material other four distinct crosstalks can be seen (Fig. S2).

**Figure 6:**
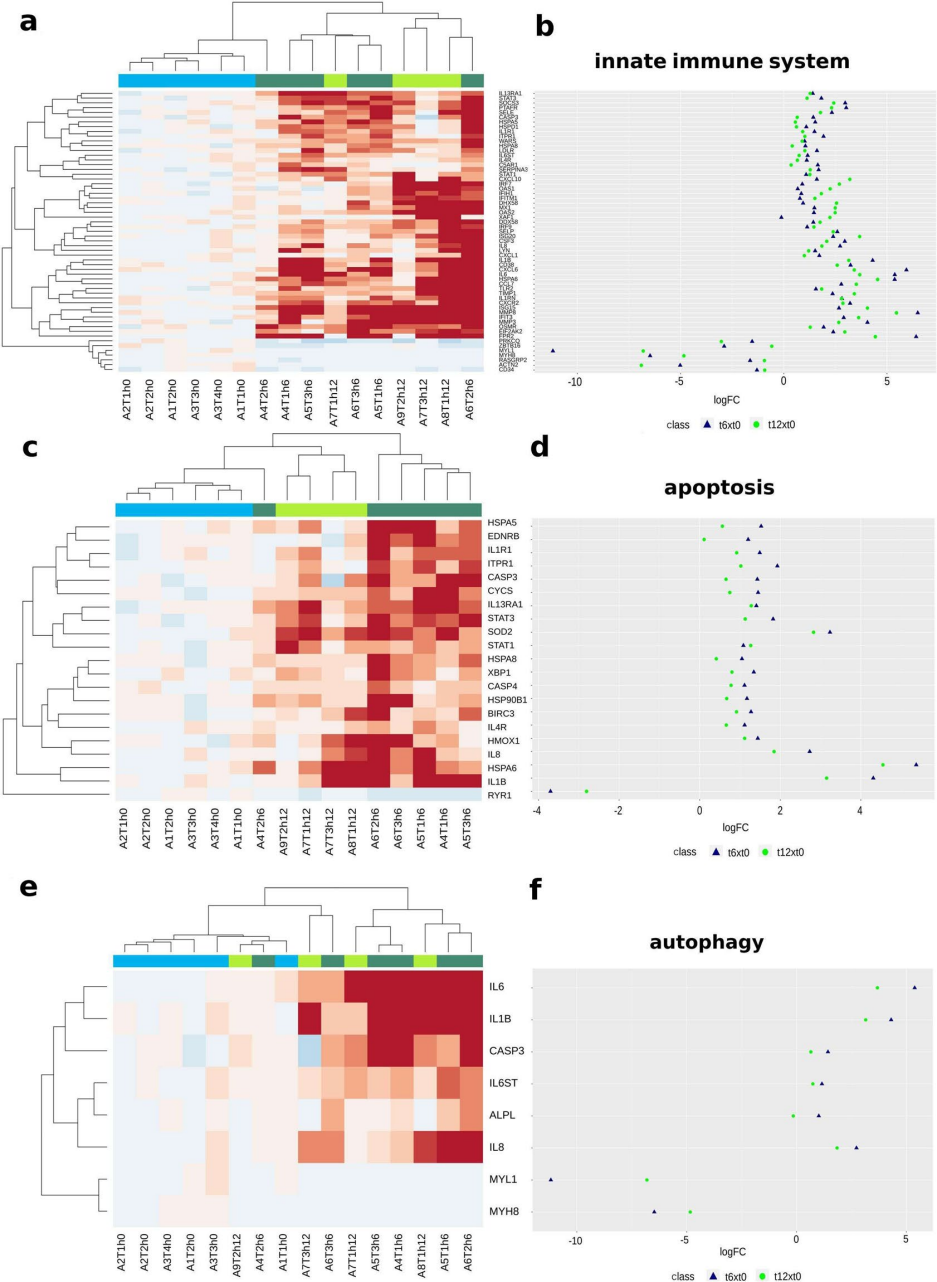
Heatmap and dotplot for the innate immune system (**a,b**), apoptosis (**c,d**), and autophagy (**e,f**). There are 59 genes related to the Innate Immune System and most of them were upregulated. Here, hierarchical cluster analysis shows 6 h samples mixed with 12 h samples, but completely apart form 0 h. Genes like *CXCL10, IRF7, OAS21, IFH1, IFITM1, MX1, OAS2, XAF1, DDX58, IRF9*, and *ISG20* are more upregulated at 12 h than 6 h, meaning that RLR and OAS pathways responses are increasing in time. Genes like *MYL1, MYH8*, and *ACTN2* are downregulated, the first two with higher modulation at 6 h, and the latter at 12 h. Apoptosis split very well the 3 time points, besides sample A4T2h6 sample, related to 6 h, is closer to 12 h. Many apoptotic genes were upregulated, except *RYR1*, with a high modulation for *SOD2*, *HSPA6* and *IL1β*. Genes related to autophagy could not enrich the “autophagy pathway”, but *IL6, IL1β, CASP3, IL6ST, ALPL*, and *IL8 were* upregulated DEGs, and *MYL1* and *MYH8* were downregulated DEGs.

All five crosstalks showed a strong interaction between genes related to different biological functions; additionally, there were genes that participate simultaneously in different functions (pleiotropy). Only autophagy was not seen, possibly because it is not based on a phenomenon of transcriptional modulation. Crosstalk analysis between Innate Immune System x Apoptosis x Autophagy can be seen in Fig. 5, and respective enriched gene modulations in Fig. 6 (other crosstalks can be seen in Fig. S2, and respective heatmap, dotplot, and spaghetti plots in Fig. S3). There is a stronger interaction between genes for 6hx0h compared to 12hx0h. All circlize plots have more genes and are more connected for 6hx0h compared to 12hx0h, demonstrating a strong response at 6 h, diminishing at 12 h.

#### CEMiTool/WGCNA coexpression analysis

Performing coexpression of genes modulated by Amblyomin-X treatment using CEMiTool, yielded 33 modules named M1 to M32, and an additional one called not-correlated (Table S8). Enrichment analysis was calculated on these modules using KEGG, Reactome, WikiPathways and Gene Ontology (GO), which resulted in a predominance of signaling pathways related to muscle, interleukins, and response to virus infection. Interestingly, some of the genes enriched for these pathways were positioned just after the DEG definition threshold.

For instance, 7 genes (*ISG15*, *CASP10*, *CASP8*, *TNF*, *DHX58*, *TRAF3*, and *IRF7*) were found, related to KEGG RIG-I-like receptor signaling pathway/RIG-I-like Receptor Signaling WP3865 at M13, although only 3 (*ISG15*, *IRF7* and *DHX58*) out of those 7 were DEGs for 12hx0h and only one (*ISG15*) for 06hx0h. Since these genes are clustered in the same module, they showed the same expression pattern.

In this same module M13, seven genes (*IRF1, CASP10, CASP8, TNF, CDKN2A, TRAF3*, and *IRF7*) were related to apoptosis and suggesting that Amblyomin-X has triggered this pathway. *HSH2D*, coding for a protein that may be a modulator of the apoptotic response through its ability to affect mitochondrial stability^38^, was one of the main intramodular hubs in this module. Another intramodular hub in module M13 is *ORM1*, coding for a member of the lipocalin family of proteins, with a function in modulating the innate immune system in events of acute phase inflammation, and known to bind synthetic drugs^39^. *ORM1* is also a DEG for 06hx0h and 12hx0h comparisons. The interaction hub in module M13 was *SMURF1*, which promotes ubiquitination and subsequent proteasomal degradation of *MAVS*^40^ and plays a role in dendrite formation by melanocytes^41^.

In module M9, 13 genes (*H2AFZ, HLA-B, HLA-C, HLA-F, PSMA7, PSMB1, PSMB2, PSMB3, PSMB6*, *PSMC3, PSMC4, PSMD8*, and *UBE2D2*) were found, enriched by WikiPathways for “Proteasome Degradation” (WP183 from WikiPathways), and 16 (*ASB1*, *ASB8*, *KEAP1 PSMA7*, *PSMB1*, *PSMB2*, *PSMB3*, *PSMB6*, *PSMC3*, *PSMC4*, *PSMD8*, *RNF138*, *RNF217*, *UBE2D2*, *UBE2M*, and *TRAF7*) enriched by Reactome for “Antigen processing: Ubiquitination & Proteasome degradation”. None of which are DEGs, but still following the same pattern of increase in expression over time. We expected such a phenomenon with Amblyomin-X treatment^19^.

In module 9, the major intramodular hub is *PRELID1*, involved in the modulation of the mitochondrial apoptotic pathway by ensuring the accumulation of cardiolipin (CL) in mitochondrial membranes. One of the interaction hubs in this module is *UBE2M*, one of the ubiquitin-conjugating enzymes. The encoded protein is linked with the ubiquitin-like protein *NEDD8*, which can be conjugated to cellular proteins, such as Cdc53/Culin.

#### Cellular interaction partners of Amblyomin-X

Identification of cellular interacting partners is critical to understand the initial process originated by Amblyomin-X, resulting in modulation of pathways at the later stage of the route. Therefore, we obtained the equine melanoma cellular interactomics profile for Amblyomin-X by co-precipitation. Multiple potentially interacting proteins were identified in eluent fraction that bound to immobilized Amblyomin-X. The list of proteins is shown in Table S9, including unique identifiers and the number of peptides. Interestingly, we found proteins related to biological effect previously reported in Amblyomin-X studies, such as apoptosis, mitochondrial dysfunction, regulation of cell migration and protein clearance, which includes cytochrome-c, plasminogen, actin cytoplasmic 1, fibronectin, heat shock protein HSP 90-alpha, E3 ubiquitin-protein ligase Mdm2, mitochondrial superoxide dismutase, and vimentin^15,18,42,43^. Furthermore, we identified immunogenic proteins such as Toll-like receptor 2 (*TLR2*) and T-cell surface antigen CD2 (*CD2*) as interacting partners of Amblyomin-X.

#### Validation of RNA-Seq results through qRT-PCR

This study selected 56 DEGs related to innate immune response, apoptosis and inflammation for validation with qRT-PCR. Next, these genes were classified into 3 groups, according to the median expression: highly expressed (one of the median expressions greater or equal to 50 CPM), moderately expressed (not lowly neither highly expressed) and lowly expressed (all expressions less than 5 CPM) (Table S10). Forty-two (42) out of these 56 genes were chosen for qRT-PCR validation (Fig. S4). The correlation between qRT-PCR LFC for 6hx0h and RNA-Seq LFC for 6hx0h (Table S11) was 80.2%, and the correlation between qRT-PCR LFC for 12hx0h and RNA-Seq LFC for 12hx0h was 81.5% (Fig. S5).

## Discussion

Amblyomin-X treatment reduced equine melanoma tumor size, after which we investigated modulated genes and enriched pathways. The study considered as confounding factors: the pathways related to needle tissue injury as wound healing, the increase in tumor volume as hypoxia, and the vein injury as vascular endothelial cell damage. Although real, these processes were probably not the reason for the decrease in tumor volumes since many tumors treated with vehicle did not show a decrease in size.

Our results validated previous uses of Amblyomin-X against several tumor cell lines including skin melanoma^15–18,42^ like ER-stress, Mitochondria-stress, and Apoptosis. It is important to note that the transcriptome results presented herein are the mean cell expression values in the tumor’s core, a complex system composed of many distinct normal cells surrounding tumor cells. It is also necessary to observe that pleiotropic effects can deceive the expression of some DEGs related to these functions. For instance, Actins and Calcium ion play an important role in pathways such as Endoplasmic Reticulum Stress (ER-stress), Cytoskeleton remodeling, and Muscle contraction, and IL-8 plays a role in endothelial damage and inflammation response. Since we are interested in the orchestrated responses between Immune System, Inflammation, Apoptosis, ER-stress and Cytoskeleton remodeling, any actin or calcium ion release, belonging only to muscle processes, would confound our inferences. Taken the above into consideration, we presented the most statistically significant genes and pathways modulated by Amblyomin-X (see also supplementary material).

**Figure 7:**
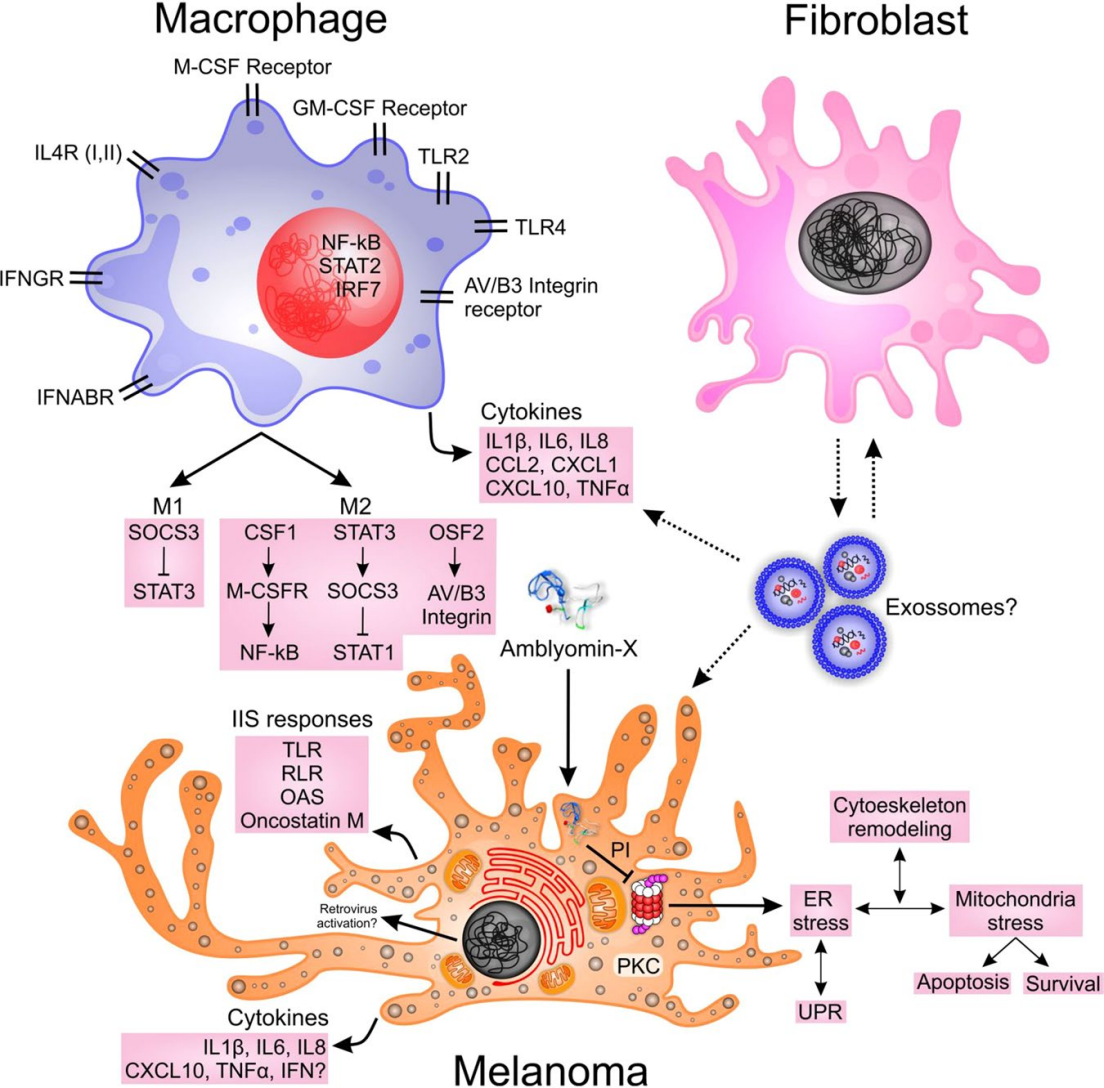
Schematic overview of a simplified tumor environment model. Melanoma, Macrophage, and Fibroblast (stroma) are possibly the main cells interacting through cytokines, exosomes, and other signaling proteins. Herein, we postulate that some Melanoma cells enter in ER-stress state because Amblyomin-X was internalized, next mitochondria stress and cytoskeleton remodeling occur, and finally, apoptosis and survival duel are the final outcomes. Concomitant, IIS pathways are activated in melanoma cells, first by the RLR responses, possibly sensing RNA molecules originating from fibroblast exosomes^44^ or being transcribed from endogenous retroviral elements^45^. IFN type I and NOXA transcriptions were not seen, but many cytokines (*IL1β, IL-6, IL-8, IL12, IP10*, and *TNF-α*) were DEGs, and their proteins were likely to have been produced and released to extracellular medium. Many macrophage signaling pathways were enriched, and their transcribed cytokines are *IL1β, IL-6, IL-8, CCL2, CXCL1, CXCL10 (IP10)*, and *TNF-α*, all DEGs. Possibly the production of some of these proteins results in M1 and M2 polarization, since *STAT1, STAT3*, and *SOCS3* were also DEGs. These macrophage cytokine releases should lead to many processes, including a crosstalk to Fibroblast, but mainly, the feedback to RLR pathway and the inflammatory response, see text. All these inferences were made according to Metacore algorithms, references, and database.

Figure 7, a simplified model of the tumor microenvironment, shows the Amblyomin-X molecule in the extracellular medium. That allows us to hypothesize on how some pathways are interrelated and evolve in time. Amblyomin-X is able to enter cancer cells that expose phosphatidylserine on the external membrane, entering by endocytosis, and promoting ER-stress through the proteasome inhibition (PI) or other unknown processes. Next, a series of related phenomena are observed, like Unfolded Protein Response (UPR), Mitochondria-stress and Apoptosis. We also hypothesize that in parallel or due to the cell stress, a second phenomenon of exosomes transport^44^ occurs between these cells or, less likely, the activation of retrovirus transcription in cancer cells^45^. Both processes release viral RNA that interact with viral RNA sensors (*RIG-I, LGP2, MDA5*, and *DDX60*) activating the RLR pathway, which has three outcomes: a) apoptosis through *PMAIP1* (*NOXA*); b) interferon transcription, through *TBK1, IRF3*, and *IRF7;* and c) cytokines transcription (*IL1β, IL-6, IL-8, IL12, IP10*, and *TNF-α*) through *NF-κB*. Related to the NF-κB path, *IKB* must be degraded by the proteasome in order to *NF-κB* to migrate to the nucleus, however, we just hypothesized that the proteasome was inhibited. Taking this into account, the transcription of these cytokines should decrease in melanoma cells. However, other cells, like macrophages, will be able to transcribe these cytokines after RLR signaling activation. Therefore, some melanoma cells will be unable to sustain the cytokine transcription, but paracrine signaling will be present and RLR sensors transcription will be boosted, as well as the inflammatory response, and probably M1 and M2 polarization starts. As mentioned before, these signaling crosstalk should increase the inflammatory response, and also *TREM1* and IL-6-induced acute-phase response (downstream *ORM1, SERPINE1, CXCL1, and C3)* pathways will be activated.

Interesting evidence was the simultaneous activation of different innate immune system pathways, increasing the expression of many DEGs related to RLR, OAS, TLR and Oncostatin-M pathways. Observing Fig. 6a,b, most of the DEGs related to IIS have a maximum fold-change at 6 h, decreasing their expressions at 12 h, but still remaining DEGs; besides *CXCL10, IRF7, OAS1, IFIH1, DHX58, MX1, OAS2, XAF1, DDX58, IRF9, ISG20, CCL7* (or *CCL2*, ortholog), *TIMP1, ISG15, ZBTB16, MYH8, RASGRP2*, and *CD34* increasing their expression after 6 h, perhaps boosting the IIS response. In Fig. 6c,d, most of the genes related to apoptosis have also a higher expression at 6 h; therefore, we cannot infer causality between IIS and Apoptotic genes and pathways.

We hypothesize that *PKR (EIF2AK2)* is a central hub related to the innate immune system pathways, supporting the transcription of inflammatory cytokines. As mentioned before, macrophages continuously induce the transcription of *IL1β, IL-6, IL-8, CXCL1*, *CXCL10*, and *TNF-α*, the last of which was moderately expressed. *CXCL10* probably stimulates monocytes, NK cells, and T-Cells or plays a role in modulating adhesion genes. Interferons type 1, *IFNα* and *IFNβ*, should be transcribed through one of the RLR pathway-branches - via *TBK1, IRF3*, and *IRF7 -* but we could not observe any transcript expression^46,47^. It is also important to highlight that *TMEM173* (*STING*) was moderately expressed and not modulated, and *PMAIP1* (*NOXA*), an important gene related to the Bortezomib pathway, was not transcribed. These results demonstrated that Amblyomin-X follows different routes.

Another important pathway was Cytoskeleton remodeling, enriched for 6hx0h. Interestingly Öhman *et al*.^48^ described the crosstalk between many RLR proteins and Actin and Tubulin proteins close to the mitochondria. In our results *ACTN1* and *ACTR3* are upregulated DEGs; *ACTA1* and *ACTN2* are highly downregulated DEGs; and regarding Tubulin family, *TUBB* and *TUBB6* are upregulated DEGs.

“Oncostatin M signaling via JAK-Stat” or “Oncostatin M signaling via MAPK” pathways are related to the ligand *OSM* (Oncostatin M) and their receptors. *OSM* is a cytokine member of the leukemia inhibitory factor/oncostatin-M (*LIF*/*OSM*) family and a growth regulator with important roles in inflammation and tumor growth inhibition. *OSM* effector was not a DEG showing low expression, but its receptors were DEGs (*OSMR* and *OSM receptor*). Downstream, *TIMP1*, a Metalloproteinase inhibitor, seems to inhibit *MMP1* trying to stop ECM remodeling; *CCL2* (or *CCL7*, its horse ortholog gene) increases inflammation signal and *SERPINE3*, a plasma protease inhibitor, probably regulates inflammatory response.

### Amblyomin-X modulates ER-stress, mitochondria dysfunction, and apoptosis

Many apoptotic signals were high at 6 h (*CASP3*, *CASP4*, and *CYCS*) and their expression lowered at 12 h. Survival signals showed the same behavior (*BIRC2*, *BIRC3*, *ATF6*, and *SOD2*); besides; *SOD2* (up for 6hx0h, and up for 12hx0h), was the only pro-survival signal which is upregulated at both times. At 6 h cytochrome-c (*CYCS*) was upregulated, which shows a possible mitochondrial damage. Calpain 2 (*CAPN2*) and *HSP60*, the latter was a DEG, and *BAX* (verified in previous *in vitro* experiments) support this hypothesis. We assume that ER-stress shows a peak close to 6 h since *GRP78*, *IP3R1*, *ATF6*, *XBP1*, *PDIA6*, and *HSP90B1* were upregulated at this time. Since the treatment can kill some cells and allows others to survive, we hypothesized that it also activates immunogenic cell death (ICD) mechanisms via ER-stress with the release of *HMGB1* and *CALR* (calreticulin), an important DEG at 6 h, which can be found close to ER, in the inner side of the cell membrane, and also on the external side. According to Jheng *et al*.^49^, we could map autophagy upstream signals related to ER-stress with the following genes: *CASP4*, *CASP12*, *JNK*, *ATF6*, *CHOP*, *ATF4*, *EIF2AK3* (*PERK*), *EIF2A*, and *GADD34*. Many of them were well expressed, but not modulated. Therefore, autophagy may start later, since it was not enriched, or may even not start (see Fig. 6). Since Amblyomin-X can inhibit the proteasome machinery, some proteins accumulate nearby ER and new chaperones were transcribed (*HSPD1* (*HSP60)*, *HSPA4* (*HSP70)*, *HSP90AA1* (*HSP90*)) resembling what the UPR, an important mechanism belonging to ER-stress pathway. HMGB1-RAGE signaling pathway was also enriched, indicating that it is possibly another effector inducing the transcription of *IL6*, *IL1β*, *CXCL8 (IL8*), *IL1RN*, *SERPINE1* (*PAI1*) and *CHGA* (Chromogranin A). The data was supported by the interactomics profile of Amblyomin-X, in which the binding partners identified were proteins such as cytochrome-c, plasminogen, actin cytoplasmic 1, fibronectin, heat shock protein HSP 90-alpha, E3 ubiquitin-protein ligase, mitochondrial superoxide dismutase, and vimentin.

In conclusion, Amblyomin-X is an antitumor molecule because it acts on multiple cellular targets to induce tumor cell death. It selectively enters only cancer cells via endocytosis, binding to the external membrane attracted by phosphatidylserine affinity, and thereafter, inside the cell, modulates its microenvironment. Amblyomin-X activates the ER- and mitochondria-stress pathways followed by apoptosis and survival responses. There is also a crosstalk between proteasome inhibition and ER-stress that must be further studied. The activation of the innate immune pathways inducing pro-inflammatory cytokines and chemokines was observed, but the initial cause is still unknown. Interferons type I transcription were expected to be seen, but an unknown process inhibits it. We believe that a more detailed data acquisition between 0 h and 6 h will elucidate some processes and their causes. In summary, the horse melanoma tumor model shed light on new genes, pathways, and crosstalks among them, showing to be a useful tool in which our new outcomes must be validated, in the future, in human melanoma experiments.

## Methods

The whole experiment was divided in: a) *in vivo* melanoma tumor treatment for a month and follow-up with clinical lab tests for up to five months; b) excision of *in vivo* melanoma tumors for histology; c) e*x vivo* excision melanoma tumors after 0 h (control), after 6 h treatment, and after 12 h treatment followed by interactomics and proteomics (Fig. S6).

### Experimental conduct and ethical compliance

Horses were housed and treated on the Sao Joaquim farm (Araçariguama, SP, Brazil) of the Butantan Institute (Sao Paulo, SP, Brazil). All animal procedures were previously approved by the internal ethics committee of the Butantan Institute (CEAUIB/BUTANTAN, protocol numbers 1999061115 and 8644050716) and were conducted in compliance with Good Clinical Practice guidelines (European Union Committee for Veterinary Medicinal Products), with the Brazilian National Council of Animal Experimentation Control (CONCEA, DBCA v.12/2016), and with the Brazilian Ministry of Agriculture, Livestock and Supply (MAPA) regulations. Animal husbandry was in compliance with the requirements of national legislation and local animal welfare guidelines (Brazilian Federal Law N° 11794/08). These studies were conducted under veterinary supervision, with veterinary attention available at all times.

### Expression and purification of recombinant Amblyomin-X

Recombinant Amblyomin-X was produced using an *E. coli* expression system as described previously^50^.

### *In vivo a*nimal experimental conditions and tumor treatment

Four male gray horses were the subjects used in the *in vivo* experiments herein studied. The horses, aged between 10 and 20 years, and weighing between 320 and 430 kg, were housed in stables with access to food (ration and green) and water *ad libitum*. Each animal was submitted to a general clinical exam before its inclusion in the experiment. Tumor sizes were recorded and melanoma lesions, which appear spontaneously at the perianal and ventral tail regions, were mapped. Tumor dimensions were measured with a caliper and tumor volumes calculated by approximating a sphere volume, *i.e*., using the formula for sphere volume = 4/3 π x radius 1 x radius 2 x radius^3^ (mm^3^)^51^. Deeply infiltrating tumors were rejected for treatment due to possible measurement errors. Volume measurements and excised tumor weight were used to estimate the mean tumor density value, which in turn were used to calculate the Amblyomin-X doses as 1 mg/Kg of tumor mass.

On 4 animals, we selected about 9 tumors, of which 3 were injected with Amblyomin-X every 3 days over a period of 28 days; 3 were injected with the same volume of PBS as a vehicle as often; and 3 were not treated (Table S12). Some animals had only 8 tumors available, of which we selected 3 for treatment, 2–3 for control, and the remaining 2–3 for the vehicle.

### Clinical evaluation

Body weights were recorded immediately before treatment and then after every 15 days until the end of the experiment, using a load-bar scale (TOLEDO^®^ MGR-4000). Blood samples were collected from the jugular vein for hematological and biochemical analysis at time zero and after every 3 weeks until the end of the protocol. Hematological and biochemical parameters were obtained using automated analyzers (BC-2800Vet, Midnray and Cobas-Mira Plus, Roche, respectively). Biochemical parameters included urea, creatinine, TGO (AST, aspartate aminotransferase), GGT (gamma-glutamyl transferase), BT (total bilirubin), BD (direct bilirubin), BI (indirect bilirubin), FAL (alkaline phosphatase) and ALB (Albumin). Reagents used were obtained from Kovalent, Brazil.

### Tumor resection and preservation

For tumor resection, animals were sedated with 10% xylazine hydrochloride (0.53 mg/kg) and detomidine (10 µg/kg), followed by local anesthesia with 10 mL subcutaneous injection of lidocaine. The incision site was shaved and disinfected it by wiping the skin surface with 0.2% chlorhexidine gluconate alcohol, followed by spraying the local with 79^o^ GL alcohol. After the tumor had been completely removing, the cutaneous wound was sutured and cleaned daily with gentamicin sulfate solution (100 mg/mL) and application of a gentamicin based ointment (Vetagloss, Vetenil Inc., Brazil).

### Histological analysis

For histological analysis, excised tumors were fixed in 10% buffered formalin solution, dehydrated and enclosed in synthetic paraffin, followed by hematoxylin and eosin staining.

### *Ex vivo* experiment: transcriptome analysis

Nine horses were selected for the *ex vivo* experiment. Melanoma tumors, in the ventral tail, were selected for transcriptome analysis. Treated tumors were injected with a single dose of Amblyomin-X (1 mg/kg of tumor), being excised 6 hours and 12 hours after the treatment, and controls were untreated tumors being injected PBS at 0 h. For each time-point, two tumors were removed from three different animals, yielding six tumors per time-point for analysis. The transcriptomic design can be seen on Table S13.

See supplementary material documentation for RNA extraction and library preparation, Quality and filtering fastq Reads, cDNA synthesis, and qRT-PCR

### Protein interactions with Amblyomin-X

Purified and lyophilized Amblyomin-X (6 mg) was dissolved in 1 ml of 0.2 M NaHCO_3_ containing 0.5 M NaCl, pH 8.3. Proteins were immobilized on a HiTrap™ NHS-activated HP 1 ml column (GE Healthcare Life Sciences) according to the manufacturer’s instructions. After this, tissue extract from untreated equine tumor samples were applied to the HiTrap™ Amblyomin-X affinity column. The column was washed with 60 ml of 20 mM Tris-HCl buffer, pH 8.3, to eliminate any non-specific protein interaction. Bound proteins were eluted with 200 mM glycine containing 0.5 M NaCl, pH 4.0. The eluent was extensively dialyzed with 25 mM ammonium bicarbonate and dried in a speed vacuum evaporator (Thermo Scientific). Dried samples were stored at −80 °C or dissolved in 50 mM ammonium bicarbonate, containing 10 mM CaCl_2_ for MS/MS analyses. For protein identification, trypsin hydrolysates samples were injected into an EASY-nano LC system (Proxeon Biosystems) coupled online to an ESI-LTQ-OrbitrapVelos mass spectrometer (Thermo Fisher Scientific), which was operated in a positive mode of ionization using the data-dependent automatic (DDA) survey MS scanned tandem mass spectra acquisition. Peaks Studio 7.5 (Bioinformatics Solutions Inc. Canada) was employed for data acquisition, processing and analyses.

### Bioinformatics and systems biology

#### Expression analysis

In order to map and quantify transcripts, we used featureCounts from Subread package^52^ resulting in a table of gene IDs versus samples with raw read counts. Then, we calculated differentially expressed genes (DEGs) using the edgeR^53^ package. Genes with low expression (*i*.*e*., all sample groups having sum of counts less than one CPM, count per million in each) were filtered out. DEGs were calculated with the following comparisons: a) 6 h after treatment (6 h) x control (0 h); b) 12 h after treatment (12 h) x control; and also c) 12 h x 6 h, here named as 6hx0h, 12hx0h, and 6hx12h, respectively. Let define LFC as the log2 of the fold change between two conditions and FDR as the false discovery rate. DEGs were defined as genes with absolute value of LFC > = 1 and FDR < 0.05. All transcripts were mapped using horse Ensembl Gene ID, followed by BioMaRt tool^54^ mapping orthologs between horse gene IDs and human gene IDs (*Homo sapiens* reference genome GRCh38 and annotation version 89).

#### Enrichment, network, and coexpression analysis

Three different methods were used for *in-silico* enrichment analysis (Fig. S7). Pathway Enrichment Analysis (PEA) which uses Over-Representation Analysis (ORA) statistics and databases like Metacore^55^, Reactome^56,57^, WikiPathways^58^, KEGG^59^ and Enrichr^60,61^; Fast Gene Set Enrichment Analysis (fGSEA)^62^, and String-db^63^ version 11.0 enriching against KEGG, GO^64^, and pFam^65^.

For network analysis String-db, a PPI network, was used, setting the minimum score to 0.40. Gephi version 0.9.2^66^ was also applied, with DEGs as nodes and edge weights as PPI scores obtained from String-db. In addition, circlize^67^ was employed to simulate possible crosstalks between enriched pathways.

WGCNA^68^/CEMiTool^69^ was used for coexpression analysis, as an unbiased technique based on gene-gene expression correlation, setting a minimum of 20 genes per module, and merging modules with correlation coefficient above 0.75. We resorted to BioGrid database^70^, including only genes with enough reads, in order to build a PPI network for each module. In each module, intramodular and interaction hubs were respectively identified as coexpression and interaction.

## Supplementary information

Supplementary Information.

S Table 01.

S Table 02.

S Table 03.

S Table 04.

S Table 05.

S Table 06.

S Table 07.

S Table 08.

S Table 09.

S Table 10.

S Table 11.

S Table 12.

S Table 13.

S Table 14.

## Data Availability

Transcriptomic data were submitted to GEO/NCBI with the accession number GSE145292. Recombinant Amblyomin-X is patent granted (WO/2006/029492).
