# Supplementary material for: Modulation of stress and immune response by Amblyomin-X results in tumor cell death in a horse melanoma model
Source: Sci Rep. 2020 Apr 14;10:6388. doi: 10.1038/s41598-020-63275-2 (PMC7156751; doi:10.1038/s41598-020-63275-2)
Supplement: Supplementary file 1 — Supplementary Information. [file 41598_2020_63275_MOESM1_ESM.pdf]

Modulation of stress and immune response by Amblyomin-X results in tumor cell death in a horse melanoma model

Flavio Lichtenstein[1,2], Asif Iqbal[1,2], Sonia Elisabete Alves de Lima Will[1,2], Rosemary Viola Bosch[1,2], Carlos DeOcesano-Pereira[1,2], Mauricio Barbugiani Goldfeder[1,2], Roger Chammas[3], Carlos Eduardo Madureira Trufen[1,2], Katia Luciano Pereira Morais[1,2], Jean Gabriel de Souza[1,2], Renato Jose Mendonça Natalino[3], Inacio Junqueira de Azevedo[4], Milton Yutaka Nishiyama Junior[4], Ursula Oliveira[4], Francisco Ivanio Arruda Alves[1,2], Jaqueline Mayara Araujo[1,2], Aline Ramos Maia Lobba[1,2], Ana Marisa Chudzinski-Tavassi[1,2] *

[1]Laboratory of Molecular Biology, Butantan Institute, São Paulo, SP, Brazil
[2]CENTD, Centre of Excellence in New Target Discovery, Butantan Institute
[3]ICESP, Center for Translational Research in Oncology, Instituto do Câncer do Estado de São Paulo, Faculdade de Medicina da Universidade de São Paulo
[4]Laboratório Especial de Toxinologia Aplicada - CeTICS, Butantan Institute.
* ana.chudzinski@butantan.gov.br

Supplementary Material

Discussion

Simultaneous activation of different innate immune system pathways by Amblyomin-X

Soon after the drug injection, many processes could occur such as hypoxia, wound healing, external organic compound effects, drug action effects, among others. However, different innate immune systems (IIS) responses were remarkably found like: TLR, RLR, OAS and Oncostatin M pathway (OSMp). TLR and RLR downstream pathways resulted in the production of inflammatory cytokines, and also interferons[1], the last leading to the activation of OSM and OAS pathways [1-3]. Neutrophil and eosinophil responses may be confounders, since they may play a role in the initial tissue damage and also in response to the treatment. IIS is the first immune response of skin tissues against external pathogens. Peripheral skin

melanoma cells consist of many different cell types, and many of these cells can respond to external perturbations via inflammatory cytokines and transcribe other genes through autocrine and paracrine stimuli. Virus and bacteria infections trigger “Pattern-Recognition Receptors” (PRR), like TLRs but also RLR after the cell invasion. In the cytoplasm these pathogens are recognized by dead-box RLR helicases like *DDX58* (*RIG-I*), *IFIH1* (*MDA-5*) and *DHX58* (*LGP2*) having *MAVS* as a target. Also by non-RLR helicases like *DDX60* (a DEG), *DHX3, DDX9* and *DHX33* comprising *NLRP3* inflammasome as a target. Other midstream genes like *IFIT*, *NOD2, LRRFIP1* and *PKR,* having as target *NF-κB* and *CTNNB1* (β-catenin)[1], were observed. Furthermore, double-stranded RNA activates OAS genes (*OAS1*, *OAS2, OAS3*) that lead to the synthesis of 2′-5′ oligoA and RNase L activation[2]. Finally, possibly activated OSM pathway (OSMp) has as outcomes “remodeling of ECM”, “regulation of inflammatory response” (via *CCL2* and *SERPINA3*) and “growth modulation” (via *VEGFA* and *CCND1*, not DEGs) having as pathway: “Immune response: Oncostatin M signaling via JAK-STAT”.

The effect of Amblyomin-X on Inflammation

As previously mentioned, there must be an initial response increasing the expression level of many cytokines, most of them related to inflammatory pathways, like *IL1β* (produced by activated macrophages and proteolytically processed to the active form by *CASP1*), *IL-6* (a critical protein acting in acute and chronic inflammation), *CXCL8* (*IL-8*, secreted primarily by neutrophils), and *CCL2* (*MCP-1*, involved in immunoregulatory and inflammatory processes, acting as an antitumoral gene). By analyzing different enriched pathways, we could observe distinct inflammatory responses causing increased expression of the previously mentioned cytokines, besides genes like *DEFB4A* (Beta-Defensin 2, a microbicidal and cytotoxic peptide

secreted by neutrophils and regulated by inflammation). Another inflammatory pathway is *TREM1* response; “Immune response: TREM1 signaling pathway”), increasing the expression levels of *IL-6*, *CCL2* and *IL8*. Finally, TNF pathway was also enriched, although *TNF-α* was moderately regulated, being a proinflammatory cytokine secreted by macrophages and also produced downstream of the RLR pathway. Furthermore, TNF receptor *TNF-R1* was a DEG. Downstream of *TNF-R1* we observed two critical paths, one via *JUN* (*AP-1*), reaching two DEGs, *MMP3* and *IL-6*, and another via *NF-κB* complex and possibly related to important DEGs such as *IL-6*, *IL1β*, *SFK* (SRC family kinase, a proto-oncogene related to development and growth), and *IL1RN* (*IL1* receptor agonist that inhibits *IL1α* and *IL1β*, modulating the inflammatory response of the *IL1* gene family). Summarizing, inflammatory responses could be achieved, via *TREM1*, *IL1β*, *IL-6*, and possibly *TNF* and *HMGB1-RAGE* pathways. *IL-8* must also be part of the inflammatory response, but it is the biggest confounding factor in our analysis. Moreover, *IL-6* is a central hub for 6hx0h and also for 12hx0h. A part of these results was supported by interactomics profile of Amblyomin-X, where immunogenic protein such as TLR2 and CD2 were identified as binding partners.

ER-stress and Proteasome Inhibition & production

Many proteins that make up the main proteasome complex (the 20S and 26S units) started the transcription late after 12 h, enriching the proteasome pathway, through a set of none DEGs found in module M9 of coexpression analysis. This means that some cell types may have need to build new proteasome complexes. However, the pathway “Proteasome Inhibition” (PI) could not be enriched. It is worth noting that PI has already been evaluated and confirmed by multiple biochemical and cellular biological techniques [4-7]. One possible hypothesis of proteasome activity inhibition is HTT binding to 20S/26S [8-9]; and possible

consequences are: a) oxidative stress, mitochondria-stress, CASP3 (and CASP9), and also release of cytochrome-c, all very well established in our transcriptomics results.

What happens to Interferon transcription?

Type I IFNs were expected to be transcribed, but no transcription was observed. Once there are a strong midstream comprising *PKR* (*EIF2AK2*), *IRF7*, *IFIH1* (*MDA-5*), *DDX58* (*RIG-I*), and *ISG15* as DEGs at 12 h, and also *MAVS* (*IPS-1*), *TRAF3*, *TBK1*, *IRF3* as modulated genes, IFNs would probably start transcribing. Another hypothesis is the IFN transcription inhibition, most likely with post-translational modifications, as in TBK1 [10-12].

Pathways not found in transcriptomics

In addition, we were expecting certain pathways to be enriched, like endocytosis and vesicle transport pathways, but nothing could be seen. A possible hypothesis are post-translational modifications not detected by RNA-Seq, or the transcriptional machinery had no need to produce the related proteins.

Methods

RNA extraction and library preparation

Total RNA was isolated from cultured cells with Trizol (Ambion, Life Technologies) and purified with an RNAspin Mini kit (GE Healthcare) according to the manufacturer's instructions, with extended treatment with DNase I for 1 h.

RNA quality was evaluated with an Agilent 2100 Bioanalyzer RNA Pico assay. RNA was quantified by Quant-iT RiboGreen RNA reagent and Kit (Invitrogen, Life Technologies).

Messenger RNA (mRNA) was isolated and used to prepare the complementary DNA (cDNA) libraries following TruSeq RNA Sample Prep Kit V2 (Illumina, San Diego, CA) instructions. Briefly, mRNA was isolated with oligo-dT and purified. Then mRNA was fragmented by heating at 94°C (4 min) in fragmentation buffer. Double-stranded cDNA was synthesized, end-repaired and A-tailed. Sequencing adapters were then ligated to cDNA fragments, according to the manufacturer's protocol. cDNAs fragments were enriched after 15 cycles of PCR amplification. Library quality control was evaluated by the size distribution of the cDNA libraries measured by 2100 Bioanalyzer with DNA1000 assay (Agilent Technologies) and an ABI StepOnePlus Real-Time PCR System was used for quantification of the sample library before sequencing.

All 18 cDNA libraries were sequenced on Illumina HiSeq 1500 System, into a rapid paired-end flowcell in 200 cycles of 2x101 bp paired-end strategy.

Quality and filtering fastq Reads

Raw sequencing reads contaminants were removed with Bowtie version 2 2.2.5 [13]. Trimmomatic [14] was used to trim and remove reads with low-complexity and homopolymer enriched regions, poly-A/T/N tails, adapter sequences and low-quality bases, while fastq-mcf version 1.04.662 40 was used for sequencing quality control [15]. The reads were filtered out if more than 90% of reads corresponding to a homopolymer or low-complexity regions, and if the mean quality score was lower than 25 in a 15 window size. After trimming, all reads smaller than 40 bp were discarded. A quality check was performed using FastQC**.** Hisat2 [16] was used to align reads against horse (*Equus caballus*) reference genome (annotation version 89) [17].

cDNA synthesis and qRT-PCR

Gene expression levels of selected targets observed to be differentially expressed in the RNA-seq experiment were validated by qRT-PCR. PCR with 40 cycles and 1 µg of the resulting purified total RNA (without reverse transcription), using different pairs of primers for tubulin *TUBA1C* gene and Histone H3 (multiple copy gene) were used to confirm the absence of genomic DNA in all previously prepared samples. For measuring protein-coding mRNAs, reverse transcription (RT) was performed using SuperScript III (Invitrogen) according to the manufacturer instructions, followed by qRT-PCR. For all genes, oligo-dT and random primed reverse transcription were performed using 725 ng of total RNA in 20 µL of RT reaction with SuperScript III (Invitrogen), followed by qRT-PCR using 2 µL of the 10-fold diluted RT reaction in 8 µL of qRT-PCR (QuantStudio 3 Real-Time PCR System, Thermo Fisher Scientific). For assays, transcript levels were normalized to *RPL18* and represented as relative abundance using the delta Ct method [18]. Two controls for the RT step, one without primer (-primer) and the other without reverse transcriptase (-RT) were performed, followed by qRT-PCR with the pair of primers, to confirm the absence of RNA self-priming and of genomic DNA contamination in the RT, respectively. Conditions for qRT-PCR reactions were: 40 cycles of 95°C/15 sec, 60°C/1 min, using the specific primers listed in (Table S14).

References

1. Vabret, N. & Blander, J. M. Sensing Microbial RNA in the Cytosol. *Front. Immunol.* **4**, (2013).
2. Chakrabarti, A., Jha, B. K. & Silverman, R. H. New Insights into the Role of RNase L in Innate Immunity. *J. Interferon Cytokine Res.* **31**, 49–57 (2011).
3. Silverman, R. H. Viral Encounters with 2’,5’-Oligoadenylate Synthetase and RNase L during the

Interferon Antiviral Response. *J. Virol.* **81**, 12720–12729 (2007).

4. Maria, D. A. *et al.* A novel proteasome inhibitor acting in mitochondrial dysfunction, ER stress and ROS production. *Invest. New Drugs* **31**, 493–505 (2013).
5. Chudzinski-Tavassi, A. M., Morais, K. L. P., Pacheco, M. T. F., Pasqualoto, K. F. M. & de Souza, J. G. Tick salivary gland as potential natural source for the discovery of promising antitumor drug candidates. *Biomed. Pharmacother.* **77**, 14–19 (2016).
6. Pacheco, M. T. F. *et al.* Dynein Function and Protein Clearance Changes in Tumor Cells Induced by a Kunitz-Type Molecule, Amblyomin-X. *PLoS ONE* **9**, e111907 (2014).
7. Morais, K. L. P. *et al.* Amblyomin-X induces ER stress, mitochondrial dysfunction, and caspase activation in human melanoma and pancreatic tumor cell. *Mol. Cell. Biochem.* **415**, 119–131 (2016).
8. Mitra, S. & Finkbeiner, S. The Ubiquitin-Proteasome Pathway in Huntington's Disease. *Sci. World J.* **8**, 421–433 (2008).
9. Jana, N. R. Altered proteasomal function due to the expression of polyglutamine-expanded truncated N-terminal huntingtin induces apoptosis by caspase activation through mitochondrial cytochrome c release. *Hum. Mol. Genet.* **10**, 1049–1059 (2001).
10. Zhao, W. Negative regulation of TBK1-mediated antiviral immunity. *FEBS Lett.* **587**, 542–548 (2013).
11. Pfaller, C. K., Li, Z., George, C. X. & Samuel, C. E. Protein kinase PKR and RNA adenosine deaminase ADAR1: new roles for old players as modulators of the interferon response. *Curr. Opin. Immunol.* **23**, 573–582 (2011).
12. Hiscott, J. Triggering the Innate Antiviral Response through IRF-3 Activation. *J. Biol. Chem.* **282**, 15325–15329 (2007).
13. Langmead, B. & Salzberg, S. L. Fast gapped-read alignment with Bowtie 2. *Nat. Methods* **9**, 357–

359 (2012).

14. Bolger, A. M., Lohse, M. & Usadel, B. Trimmomatic: A flexible trimmer for Illumina Sequence Data. *Bioinforma. Btu170* 1–7 (2014).

15. Babraham Institute. FastQC. *FastQC* http://www.bioinformatics.babraham.ac.uk/projects/fastqc/ (2016).

16. Kim, D., Langmead, B. & Salzberg, S. *HISAT: Hierarchical Indexing for Spliced Alignment of Transcripts*. http://biorxiv.org/lookup/doi/10.1101/012591 (2014).

17. Stein, L. Genome Annotation: from sequence to biology - Review. *Nat. Rev. Genet.* **2**, 493 – 505 (2001).

18. Pfaffl, M. W. A new mathematical model for relative quantification in real-time RT-PCR. *Nucleic Acids Res.* **29**, e45 (2001).

Figures

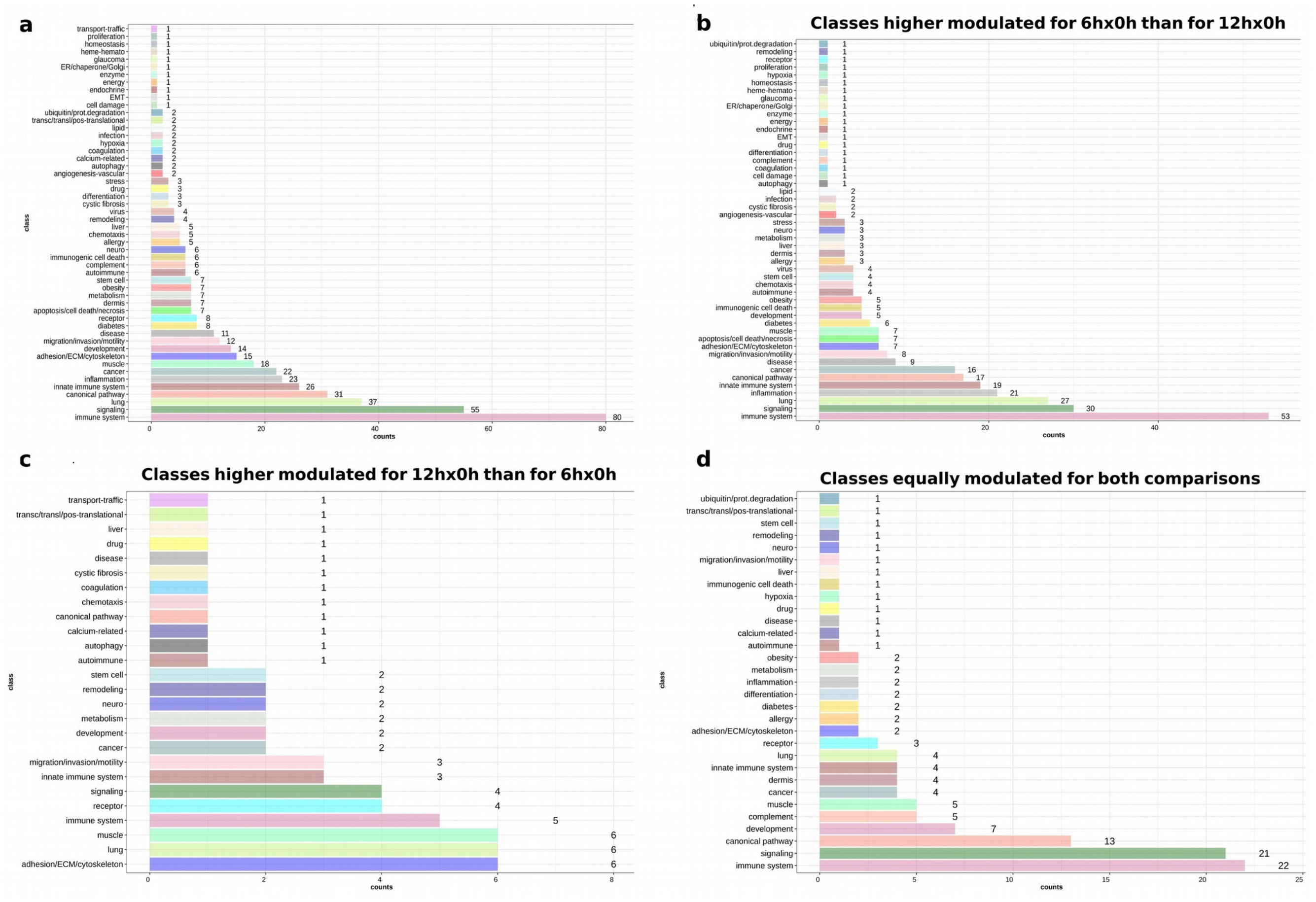

S fig. 1. Pathway classes - barplot. We can see on the upper left (a) the classification barplot counting how many pathways related to each class has been enriched. The other three barplots count pathways per classes for distinct comparisons such as (b) 6hx0h higher modulated pathway classes than 12hx0h, (c) 12hx0h higher modulated pathway classes than 6hx0h, and (d) similar modulated pathway classes.

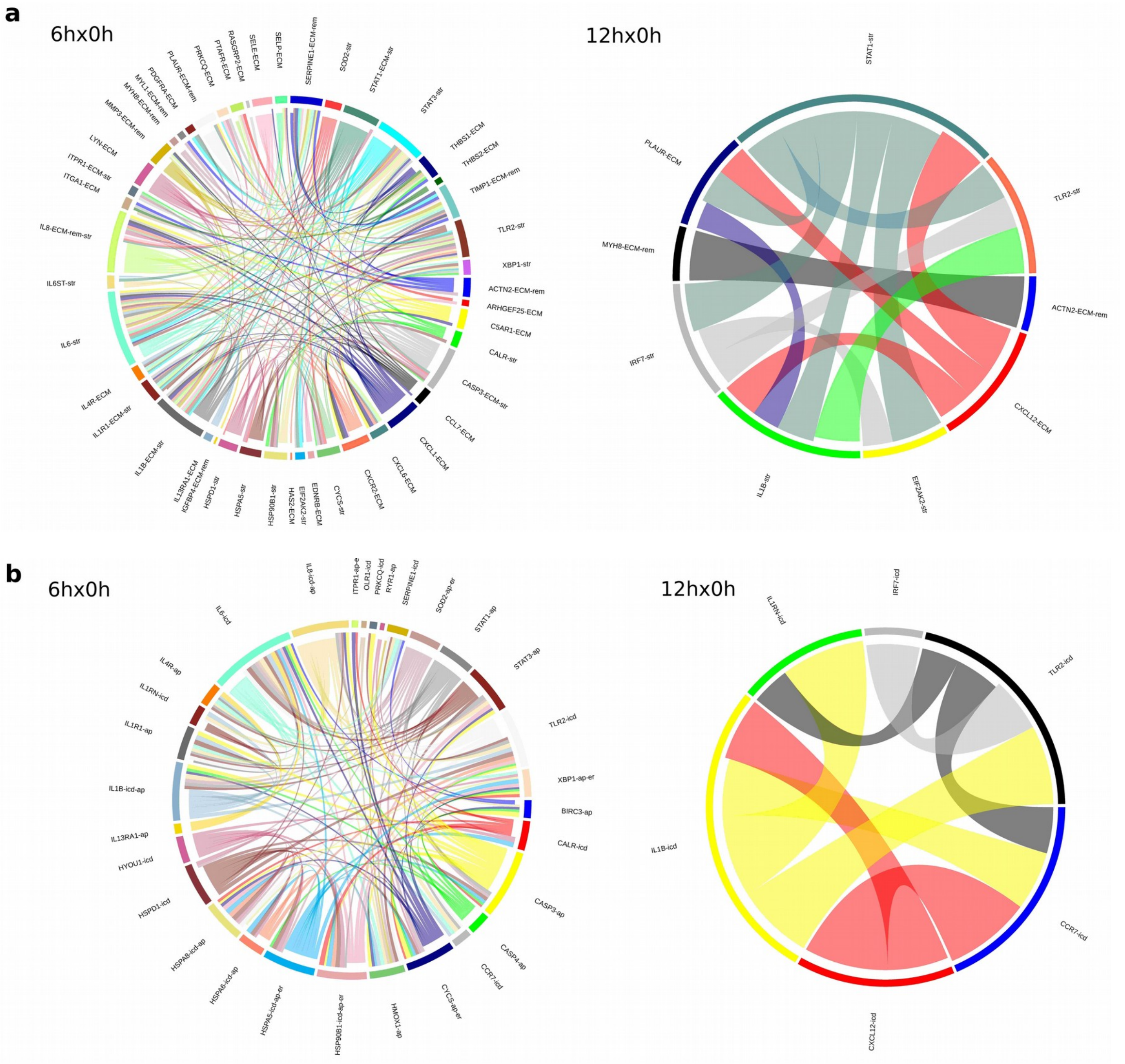

S fig. 2. Crosstalk between three pathways using circlize. Links are PPI with score greater or equal than 0.4, according to String-db. Figures (a) and (b) represent the crosstalk between DEGs related to the following pathways: ECM, remodeling and stress. Figures (c) and (d) represent the crosstalk between DEGs related to the following pathways: ICD, apoptosis and ER-stress. In each two figures, the former is for 6hx0h, and the latter is for 12hx0h. As can be seen, for 6hx0h there are more DEGs enriched in these pathways and there are also more cross-links.

a

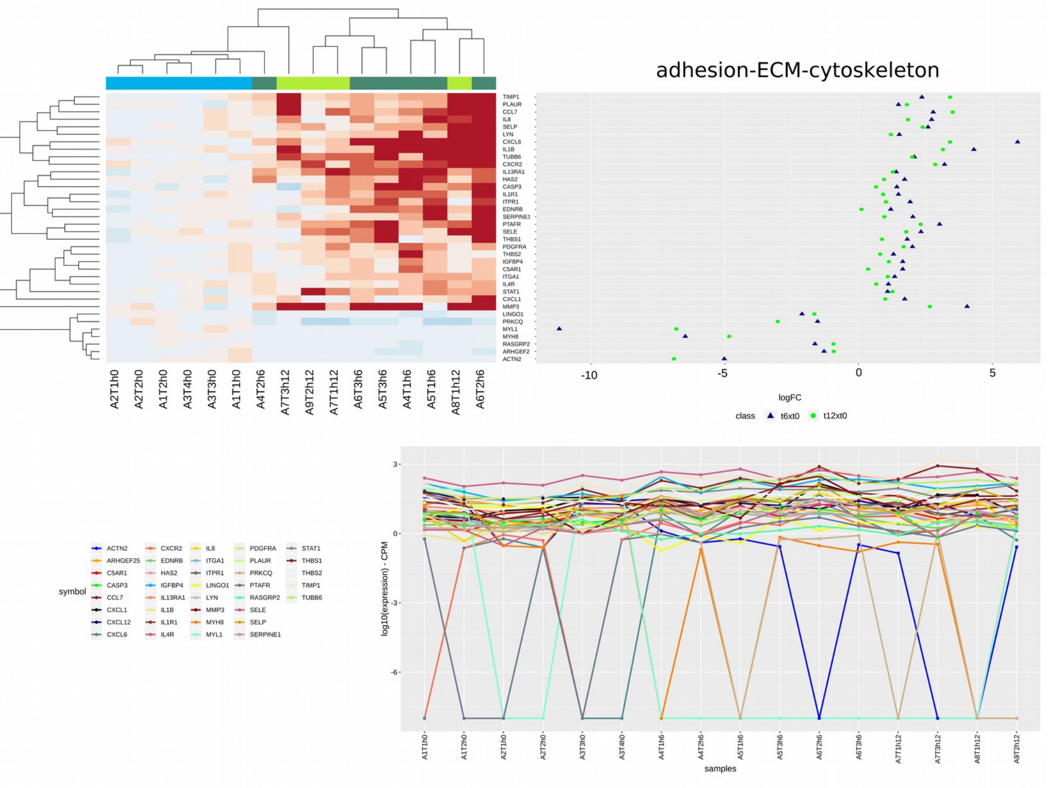

adhesion-ECM-cytoskeleton
logFC
class t6xt0 t12xt0
symbol
log10(expression) - CPM
samples

b

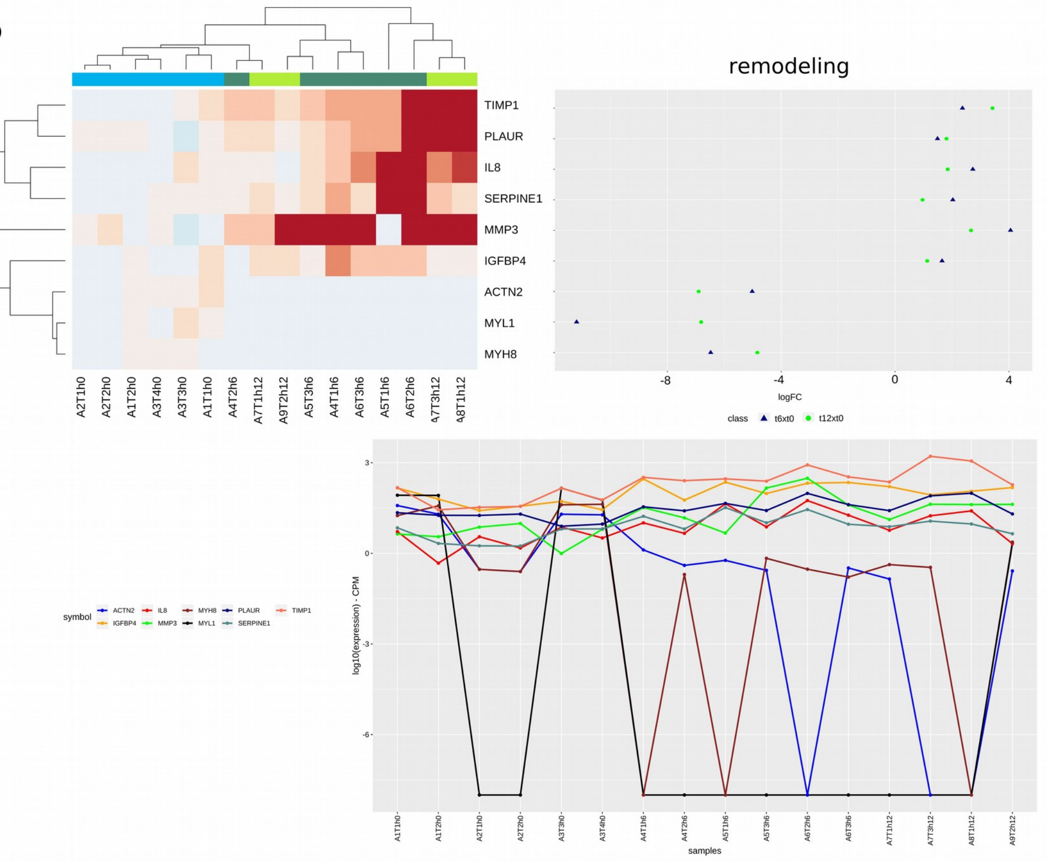

remodeling
TIMP1
PLAUR
IL8
SERPINE1
MMP3
IGFBP4
ACTN2
MYL1
MYH8
logFC
class t6xt0 t12xt0
symbol
log10(expression) - CPM
samples

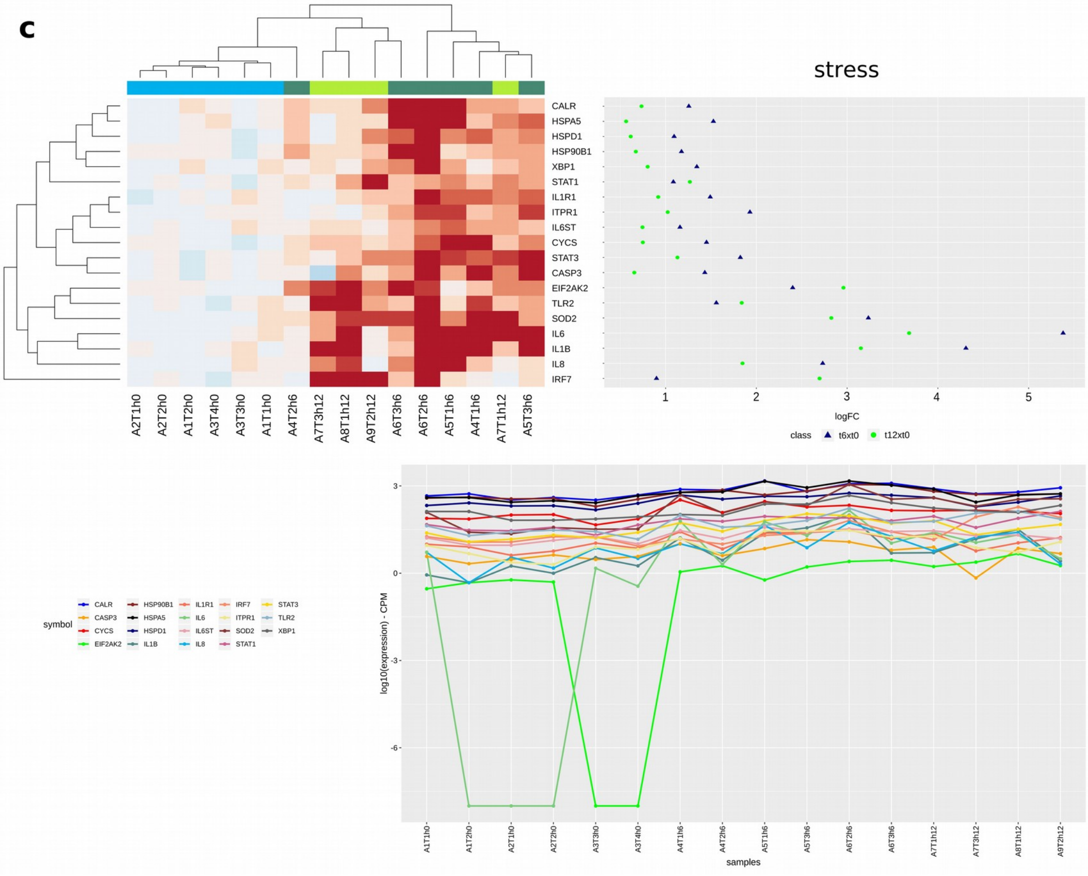
c
stress
CALR
HSPA5
HSPD1
HSP90B1
XBP1
STAT1
IL1R1
ITPR1
IL6ST
CYCS
STAT3
CASP3
EIF2AK2
TLR2
SOD2
IL6
IL1B
IL8
IRF7
A2T1h0 A2T2h0 A1T2h0 A3T4h0 A3T3h0 A1T1h0 A4T2h6 A7T3h12 A8T1h12 A9T2h12 A6T3h6 A6T2h6 A5T1h6 A4T1h6 A7T1h12 A5T3h6
logFC
class t6xt0 t12xt0
symbol
log10(expression) - CPM
samples

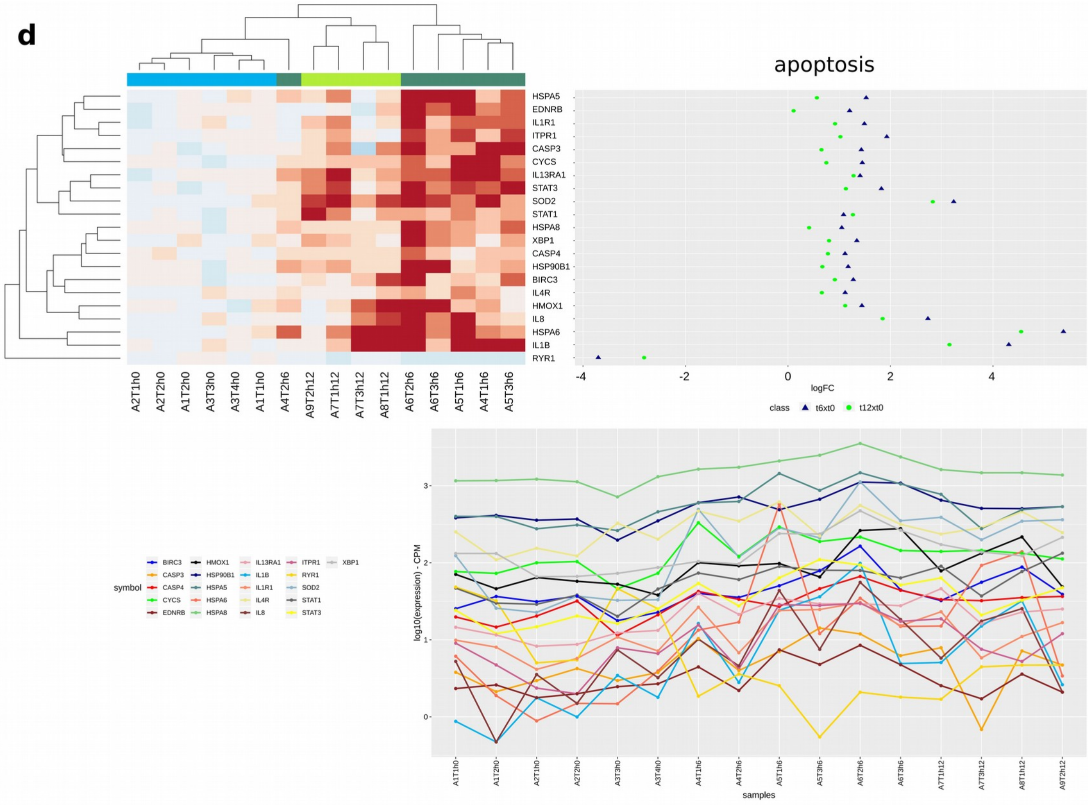
d
apoptosis
HSPA5
EDNRB
IL1R1
ITPR1
CASP3
CYCS
IL13RA1
STAT3
SOD2
STAT1
HSPA8
XBP1
CASP4
HSP90B1
BIRC3
IL4R
HMOX1
IL8
HSPA6
IL1B
RYR1
A2T1h0 A2T2h0 A1T2h0 A3T3h0 A3T4h0 A1T1h0 A4T2h6 A9T2h12 A7T1h12 A7T3h12 A8T1h12 A6T2h6 A6T3h6 A5T1h6 A4T1h6 A5T3h6
logFC
class t6xt0 t12xt0
symbol
log10(expression) - CPM
samples

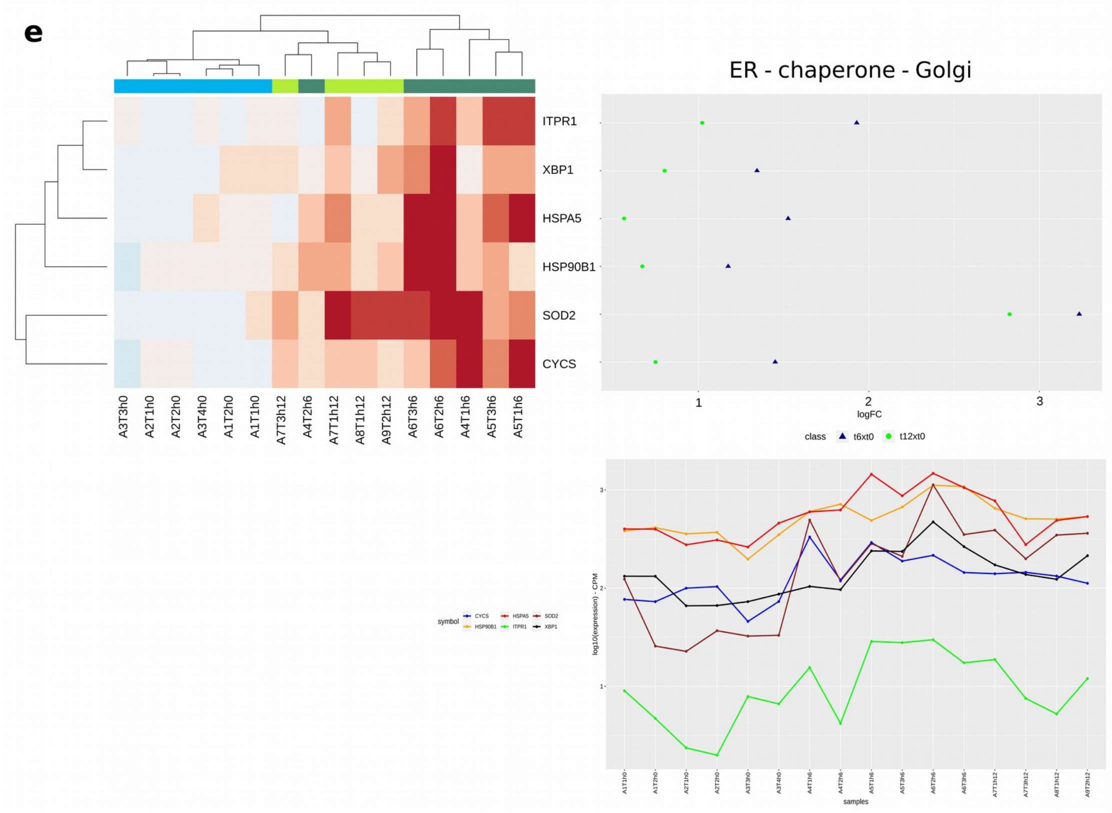
e
ER - chaperone - Golgi
ITPR1
XBP1
HSPA5
HSP90B1
SOD2
CYCS
A3T3h0 A2T1h0 A2T2h0 A3T4h0 A1T2h0 A1T1h0 A7T3h12 A4T2h6 A7T1h12 A8T1h12 A9T2h12 A6T3h6 A6T2h6 A4T1h6 A5T3h6 A5T1h6
logFC
class t6xt0 t12xt0
symbol CYCS HSP90B1 HSPA5 ITPR1 SOD2 XBP1
log10(expression) - CPM
samples

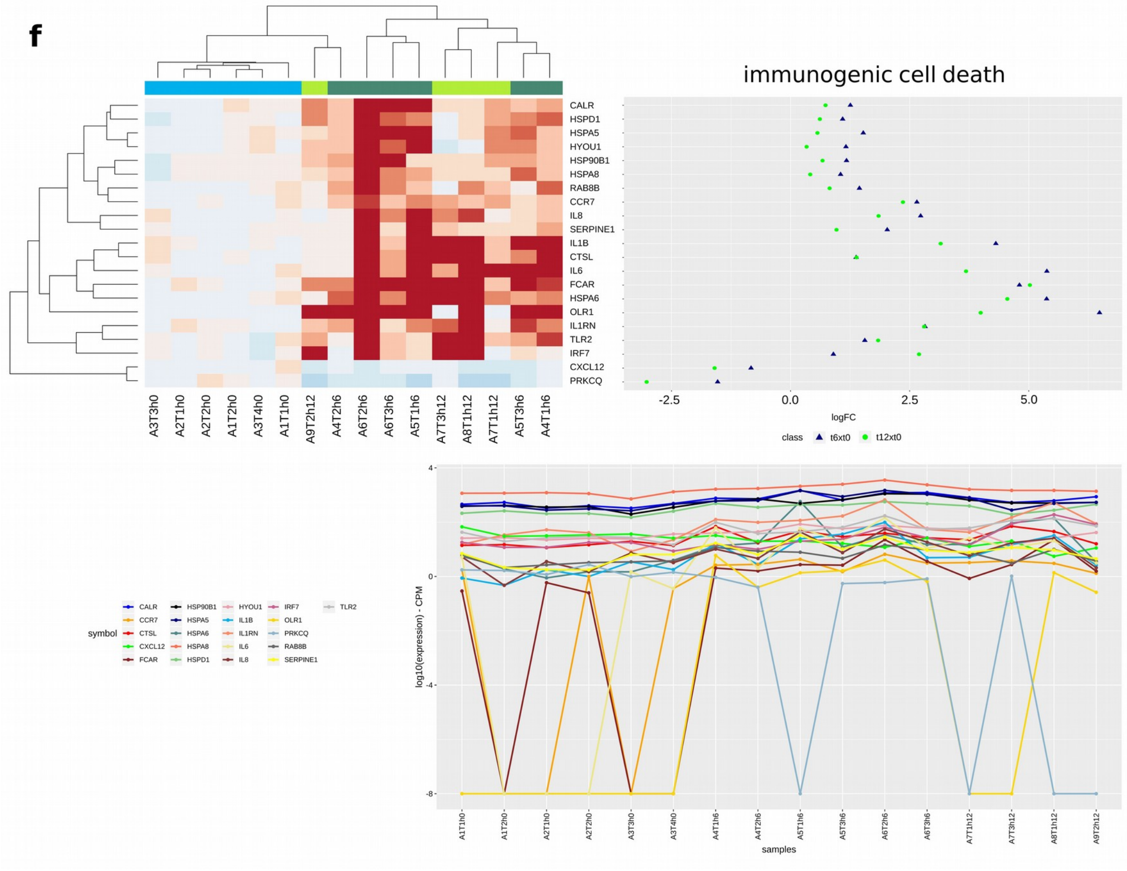
f
immunogenic cell death
CALR
HSPD1
HSPA5
HYOU1
HSP90B1
HSPA8
RAB8B
CCR7
IL8
SERPINE1
IL1B
CTSL
IL6
FCAR
HSPA6
OLR1
IL1RN
TLR2
IRF7
CXCL12
PRKCQ
A3T3h0 A2T1h0 A2T2h0 A1T2h0 A3T4h0 A1T1h0 A9T2h12 A4T2h6 A6T2h6 A6T3h6 A5T1h6 A7T3h12 A8T1h12 A7T1h12 A5T3h6 A4T1h6
-2.5 0.0 2.5 5.0
logFC
class t6xt0 t12xt0
symbol CALR CCR7 CTSL CXCL12 FCAR HSP90B1 HSPA5 HSPA6 HSPA8 HSPD1 HYOU1 IL1B IL1RN IL6 IL8 IRF7 OLR1 PRKCQ RAB8B SERPINE1 TLR2
log10(expression) - CPM
samples

S fig. 3. These figures represent the heatmap, dotplot and spaghetti plot for all pathways related to the previous crosstalks. Hierarchical analysis shows if the DEGs can split all three time points 0 h, 6 h, and 12 h. As can be seen, 0 h samples are apart from 6 h and 12 h. Some 12 h samples are close to 6 h, and others close to 0 h, the latter possibly related to the return to homeostasis state. Dotplot shows the LFC for each DEG, in blue for 6hx0h and in green for 6hx0h, as can be observed most of them are upregulated. Spaghetti plot shows how sample expressions vary for each gene. Figures 3a to 3f contain heatmap, dotplot, and spaghetti plot related to DEGs for adhesion-ECM-cytoskeleton, remodeling, stress, apoptosis, ER-chaperone-Golgi, and immunogenic cell death pathways, respectively

a 6hx0h

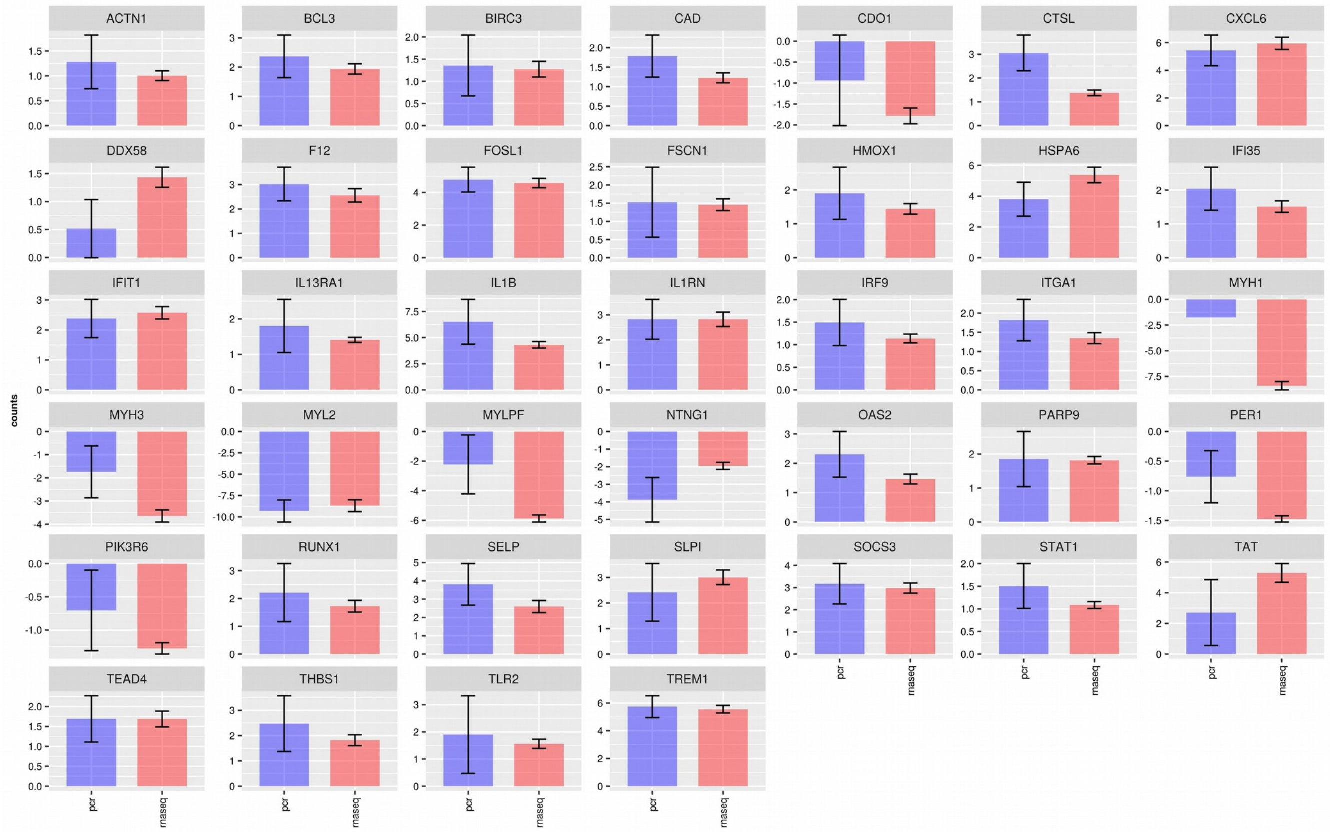

b 12hx0h

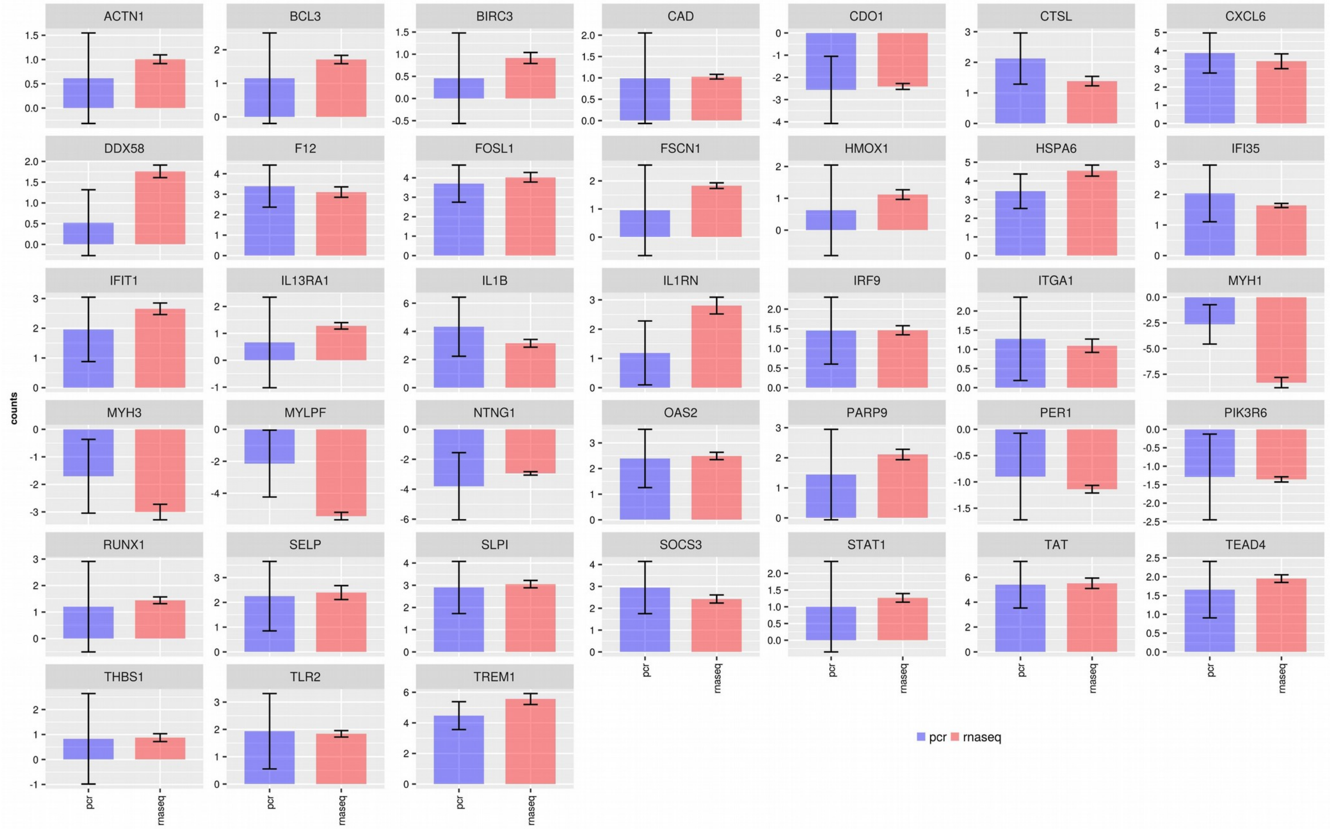

S fig. 4. Barplot for qRT-PCR validation. qRT-PCR LFC is colored blue, and RNA-Seq LFC red. Figures A are related to 6hx0h and Fig. B for 12hx0h comparison. Bar-errors correspond to one SEM calculated with n=3 for qRT-PCR, and for RNA-Seq n is equal to the number of samples (6, 6, and 4 for 0 h, 6 h, and 12 h, respectively).

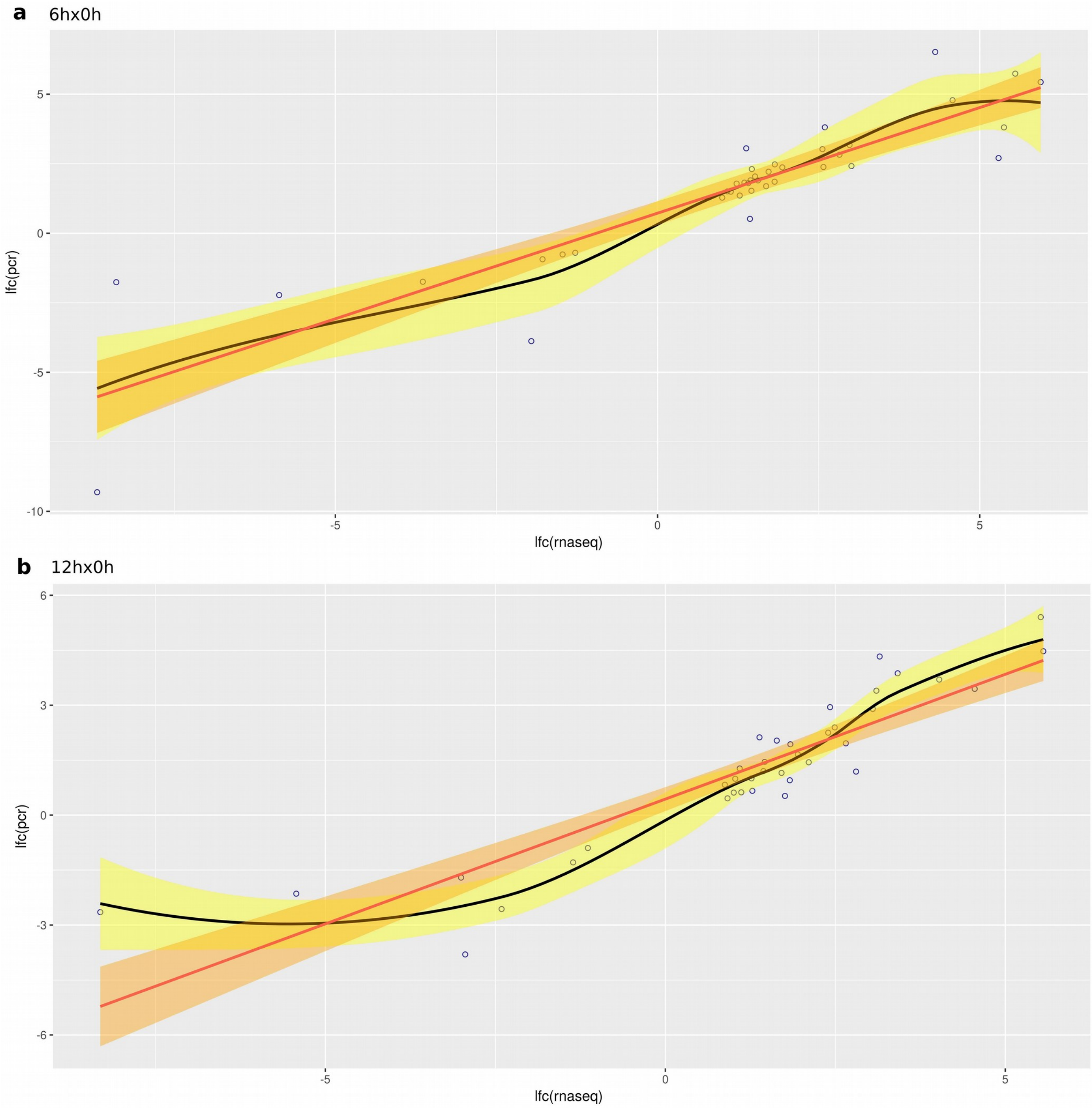

S fig. 5. Correlation between qRT-PCR LFC and RNA-Seq LFC. Correlations were calculated, in (A) R2 = .797 for 6hx0h, and in (B) R2 = 0.810 for 12hx0h.

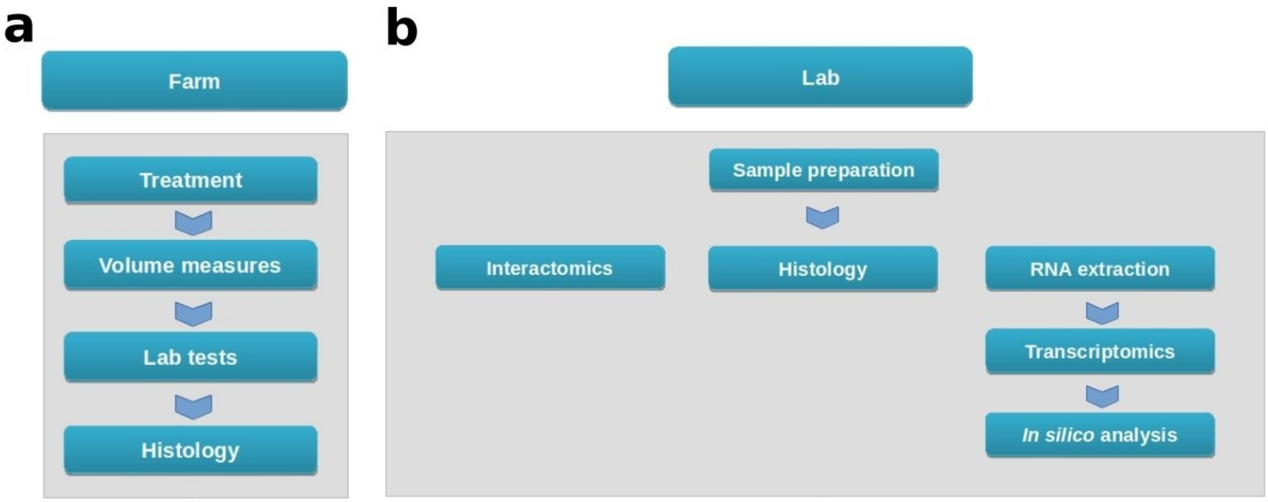

S fig. 6. *In vivo* and *ex vivo* experiments. A - on the left the *in vivo* experiment, at the farm. Horse melanoma tumors were treated with Amblyomin-X, and their progressions were controlled measuring tumor volumes, biochemical parameters, and at the end, after the tumor excision, histological analysis. B – on the right *ex vivo* experiment. Tumors were excised at 0 h, 6 h, and 12 h, after PBS and Amblyomin-X treatment, respectively. Tumors were analysed through interactomics, histology and transcriptomics. See also materials and methods.

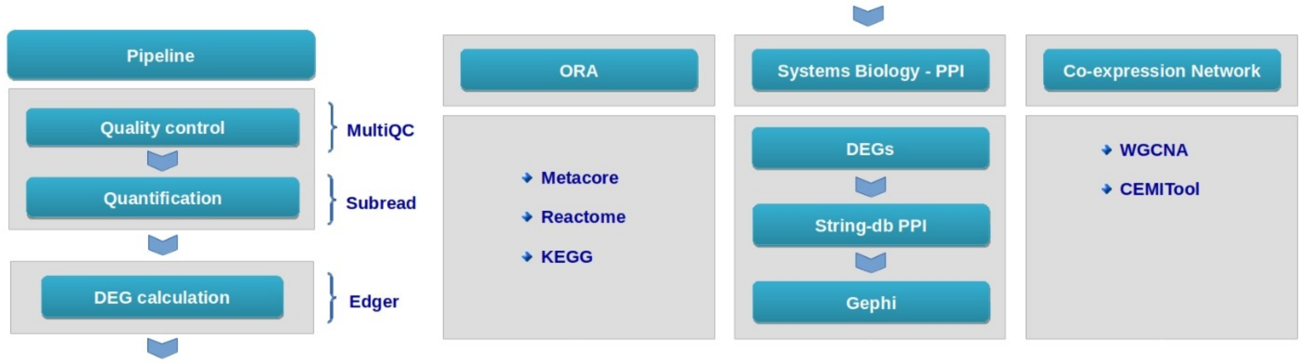

S fig. 7. Bioinformatics & System Biology pipeline. On the left the beginning of transcriptome analysis, data were obtained from Illumina HiSeq 1500, submitted to quality control, quantification and Differentially Expressed Genes (DEGs) calculation. Having the DEGs, we applied different enrichment analyses with different databases like Metacore, Reactome and KEGG. For system biology analysis we used String-db and Gephi. For coexpression analysis we used CEMiTool/WGCNA.

Tables

S table 1. Tumor volume evolution per animal (#417, #438, #795, #797). Columns are Tumor Number, Class (Treated, Vehicle, and Control), and Day Number. Each line is the Tumor Number, and each numeric cell corresponds to the mean volume in mm^3.

S table 2. Lab tests. This table has 3 sheets, the first for biochemistry (AST, Direct, Indirect and Total Bilirubin, Alkaline phosphatase, GT range, Total protein, Albumin, Urea, and Creatinine). The second sheet is RBC (RBC, Hemoglobin, Hematocrit, ACV,

ACHC, Platelets, and Plasma proteins). The third sheet is WBC (Myeloid neutrophils, Metamyelocyte neutrophils, Neutrophil Rods, Segmented neutrophils, Lymphocytes, Monocytes, Eosinophils, and Basophils). The analyzed period was from August 2015 to January 2016.

S table 3. LFC table. This table has 3 sheets for all mapped transcripts. The first sheet has the DEGs for 6hx0h comparison (# 546 genes), the second sheet has the DEGs for 12hx0h comparison (# 259 genes), and the last all mapped genes (# 14,867 genes). There are 3 columns for human gene.id, symbol and entrezid and there columns for horse gene.id_spec, symbol_spec and entrezid_spec, therefore homology can be observed. LFC, p-value and FDR are present, and all these three columns for 6hx0h and 12hx0h are present only in the third sheet. Next we can find columns such as synonyms, mean, median, SD, SEM e anova p-values.

S table 4. Enriched pathway tables. Each two sheets are enriched tables for Metacore, Reactome, String-db KEGG, and String-db GO. Each table has the pathway name, p-value, FDR, total genes, n DEGs found, DEGs found (symbol list), pathway genes (symbol list), besides other specific columns related to each database.

S table 5. Classification of differentially modulated pathways between 6hx0h and 12hx0h. Each pathway is classified according to the words in each pathway description name that may represent a function or have a gene symbol. Columns, after the class and pathway name, are delLFC.sum (sum of LFC for 6hx0h minus sum of LFC for 12hx0h, for all genes related to the pathway), p-value, FDR, and enriched

genes for 6hx0h (1) and 12hx0h (2), total of genes, how many genes could be found, how many were modulated between comparisons, nUp (number of upregulated genes), nDown (number of downregulated genes), and DEG list for up regulation, down regulation, modulation and not modulation. Positive delLFC.sum represents pathways with stronger modulation for 6hx0h, negative are pathways with stronger modulation for 12hx0h, and close to zero are enriched pathway whose high modulation is sustained throughout the experiment.

S table 6. Properties of graphs obtained using String-db KEGG and iGraph. The first sheet is for 6hx0h, and the second for 12hx0h. Genes are ordered according to k (connectivity index) and g (betweenness centrality).

S table 7. Gephi cluster analysis. The first sheet is for 6hx0h, and the second for 12hx0h. Each cluster calculated by Gephi has different colors according to the graph, Fig. 4. Columns are genes (label), degree of connectivity, weighted degree, eccentricity, closeness centrality, harmonic closeness centrality, betweenness centrality, modularity_class, and group color.

S table 8. Coexpression analysis module table. This table has the following columns: module number (M1, M2 …), gene symbol, kTotal, kWithin, kOut and Kdiff.

S table 9. Interactomic table. This table is the result from interactomic experiment. Columns are: protein ID, accession number, gene description, -10logP, coverage (%), number of found peptides, # number of unique peptides, average mass.

S table 10. qRTPCR selected genes table. This table contains the lowly, moderately, and highly expressed selected DEGs, related to innate immune response, apoptosis, and inflammation. Columns are: class, gene.id, symbol, entrezid, logFC for 6hx0h, fdr for 6hx0h, logFC for 12hx0h, fdr for 12hx0h, median for 0 h, median for 6 h, median for 12 h, and refseq.

S table 11. qRTPCR result table. Important columns are LFC: log2.ratio.cp.mean1 for 6hx0h, and log2.ratio.cp.mean2 for 12hx0h. SD and SEM are propagated according to delta Ct method. *RPL18* was used as reference.

S table 12. Equine Melanoma Tumor x Animals. This table shows, for each animal A417, A438, A795, and A797, the tumor enumeration and classes such as control, vehicle, and Amblyomin-X treatment.

S table 13. Transcriptomic design. Selected tumors from other 9 animals were used for transcriptome analysis. From each animal two tumors were selected for control (0 h, PBS), 6 h treatment, or 12 h treatment.

S table 14. Primers. Forward and reverse primer sequence, from selected genes, for qRT-PCR validation.
